# Combined Analysis of Bulk and Single-Cell Transcriptomic Data Reveals Dormancy-Associated Genes in Colorectal Cancer

**DOI:** 10.3390/ijms27125191

**Published:** 2026-06-08

**Authors:** Xiaoxi Wang, Yifan Wu, Shiyi Fang, Yubo Hu, Wenlong Li, Lingyun Zhang, Junjie Lv, Wan Li

**Affiliations:** College of Bioinformatics Science and Technology, Harbin Medical University, Harbin 150081, China; 2022020476@hrbmu.edu.cn (X.W.); wyf656218447@gmail.com (Y.W.); fangshiyilqt@163.com (S.F.); 15776801102@163.com (Y.H.); 2024020514@hrbmu.edu.cn (W.L.); 2024020515@hrbmu.edu.cn (L.Z.); lvjunjie525@126.com (J.L.)

**Keywords:** colorectal cancer, dormancy, bulk and single-cell transcriptomic data analysis, metabolic pathways, dormancy-associated genes

## Abstract

Dormancy is an important factor influencing colorectal cancer (CRC) metastasis through diverse metabolic pathways and cell types. To elucidate its molecular mechanisms, bulk transcriptomic pathway scoring was integrated with single-cell RNA sequencing of epithelial, cancer stem, and immune cells to identify CRC dormancy-associated genes (CDAGs). Twenty-three CDAGs were identified. These genes were found to play a regulatory role in dormancy by participating in metabolic processes affecting energy supply or substance synthesis. In two independent CRC cohorts (GSE41258, GSE41568), machine learning models using these genes distinguished metastatic samples with area under the curve (AUC) of 0.79–0.87. High CDAG expression was associated with better recurrence-free survival in GSE41258 (*p* = 0.005), which remained significant after adjusting for age, sex, and adjuvant chemotherapy (*p* = 0.037). The prognostic value was validated in The Cancer Genome Atlas (TCGA) Colon and Rectal Cancer for progression-free survival (*p* = 0.004). Moreover, 20 CRC dormancy-associated drugs were identified, 12 of which were reported to be associated with CRC, two with experimental evidence of inhibiting CRC metastasis or recurrence. This study provided metabolic-oriented genes for characterizing CRC dormancy, which could distinguish metastatic samples and had independent prognostic value, and offered a foundation for further development of targeted therapeutic strategies.

## 1. Introduction

Colorectal cancer (CRC) ranks as the third most prevalent and second most lethal malignancy globally. A main reason for the limited long-term survival is the high recurrence rate, especially distant metastasis. This clinical bottleneck is largely attributed to cancer dormancy—a state in which disseminated cancer cells, often remaining after curative surgery, persist for extended periods without causing clinical symptoms, yet retain the potential to reactivate and form overt metastases [1].

Tumor cell dormancy is a reversible state of quiescence (G0/G1 cell cycle arrest) that enables cancer cells to persist without proliferation while actively evading immune surveillance, undergoing metabolic reprogramming, and resisting conventional therapies [2]. Tumor dormancy arises from multiple coordinated mechanisms: (1) intrinsic cell cycle regulation leading to arrest at the G_0_/G_1_ phase, (2) microenvironmental signal transduction in response to diverse stimuli, (3) metabolic abnormalities, and (4) resistance to therapy [3]. These adaptations allow dormant cells to survive as minimal residual disease for years or even decades without rendering harm. Understanding the mechanisms that maintain stable dormancy is therefore critical for long-term disease management. Preventing dormant cells from reactivating represents one major strategy to reduce metastatic recurrence, as dormant disseminated tumor cells are a significant source of late relapse. Therefore, our study focuses on characterizing the molecular mechanisms of dormancy-associated metabolic reprogramming, with the ultimate goal of identifying therapeutic targets that either sustain dormancy as a manageable state or enable eradication of dormant cells.

Most existing studies on CRC dormancy focus on malignant epithelial cells, while some studies reported that cancer stem cells and immune cells also undergo reversible dormancy [4]. For epithelial cells, entry into dormancy is a critical determinant of metastatic latency and subsequent recurrence [5]. For CRC stem cells, there is a clear difference in the morphology of their dormant and proliferative phases [6]. Moreover, there is a dormant phenotype of immune cells in T cell acute lymphoblastic leukaemia at the bone marrow site [7]. The roles of genes related to these three cell types in cancer dormancy remain unclear, requiring further investigation.

To systematically investigate CRC dormancy at the level of its core mechanisms, we developed an integrated framework combining bulk and single-cell transcriptomic analyses. Specifically, bulk transcriptomic data from experimentally induced dormant CRC cell lines were used to discover dormancy-related metabolic pathways. The dormant state was defined at the single-cell level by identifying cells expressing dormancy markers and arrested in the G_0_/G_1_ phase. This approach identified dormancy-associated genes (CDAGs) and provided mechanistic insights into recurrence and metastasis.

## 2. Results

### 2.1. Dormancy-Related Metabolic Pathways Based on the GMMPS Algorithm

The Gene–Metabolite–Metabolic Pathway–Scoring (GMMPS) algorithm was utilized to investigate the regulatory effects of differentially expressed genes from the bulk dataset on the metabolic pathways in CRC dormancy (see Section 4 for details).

**Figure 1:**
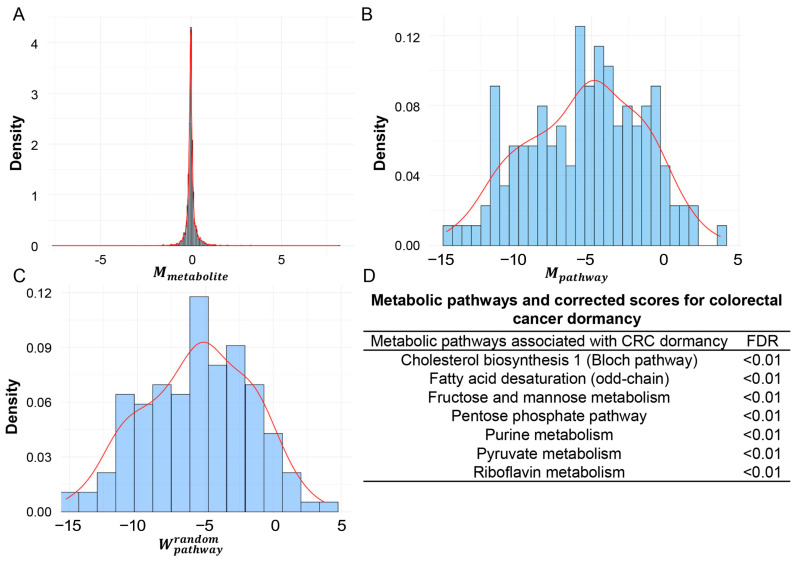
Dormancy-related metabolic pathways based on the GMMPS algorithm. (**A**) Metabolite scores according to differential genes of the bulk dataset. (**B**) Metabolic pathway scores obtained using the GMMPS algorithm. (**C**) Corrected scores of metabolic pathways. (**D**) CRC dormancy-related pathways.

Firstly, metabolite scores were obtained according to differential genes of the bulk dataset. These scores were then used to obtain metabolic pathway scores, which were further corrected (Figure 1A–C). Most metabolite scores were concentrated between −2.5 and 2.5. The majority of metabolic pathway scores were negative, as were most corrected pathway scores. A total of seven metabolic pathways were identified that met the statistical significance criteria (False discovery rate (FDR) < 0.05, top 5%) (Figure 1D and Figures S1–S6).

One of the CRC dormancy-related metabolic pathways identified was Fatty acid desaturation (odd-chain), a critical component of lipid metabolism. Dysregulated lipid metabolism activation, a central element of energetics, constitutes a hallmark feature of tumor biology. Additionally, it is metabolically linked to the Pentose phosphate pathway and Pyruvate metabolism. Pyruvate has been shown to induce quiescence in CRC cells, a phenotype consistent with tumor dormancy [8]. Dysregulation of Cholesterol biosynthesis 1 (Bloch pathway) has been proposed as a potential prognostic indicator in CRC. Plasma levels of cholesteryl esters and triglycerides of partially saturated fatty acid chains are significantly elevated in patients with advanced CRC compared to patients with early stage CRC [9], indicating the influence of cholesteryl ester metabolism on CRC progression and outcomes.

Analysis of these results revealed that these seven dormancy-related metabolic pathways were linked to CRC dormancy, providing a basis for further exploration of CDAGs.

GMMPS identified 7 dormancy-related metabolic pathways, while single-sample Gene Set Enrichment Analysis (ssGSEA) identified none. Gene Set Variation Analysis (GSVA) identified three pathways (folate metabolism, pyrimidine metabolism, and fatty acid oxidation) with significantly elevated activity in dormant vs. proliferative samples (Bonferroni adjusted *p* < 0.05), of which only fatty acid oxidation overlapped with the GMMPS set. The limited overlap between the pathways identified by GMMPS and those identified by GSVA suggests that the two approaches capture complementary aspects of metabolic regulation. In particular, GMMPS identified pathways such as odd-chain fatty acid desaturation that are not represented in standard gene-set collections. Therefore, we propose that GMMPS complements existing methods by enabling the discovery of previously uncharacterized metabolic processes related to cellular dormancy.

### 2.2. Single-Cell Landscape of Dormant-like Cells and Gene Signatures

Single-cell RNA sequencing data were analyzed to identify dormant-like cells and their gene signatures by characterizing dormancy-associated features in the tumor microenvironment and cell cycle states (see Section 4 for details).

#### 2.2.1. Cell Types

Single-cell data from primary CRC and liver metastasis with 6278 cells were clustered into 14 cell clusters (Figure S7A). Next, these clusters were annotated into epithelial and immune cells using classical marker genes (Figure S7B,C). We also performed label transfer using CellTypist, a widely used tool utilizing built-in reference profiles (Human_Colorectal_Cancer). The predicted cell types from CellTypist were highly consistent with our manually curated annotations based on the canonical markers (Figure S8A).

Dormant-like cells were identified in each cell type to facilitate subsequent gene characterization.

#### 2.2.2. Dormant-like Epithelial Cells and Gene Signatures

**Figure 2:**
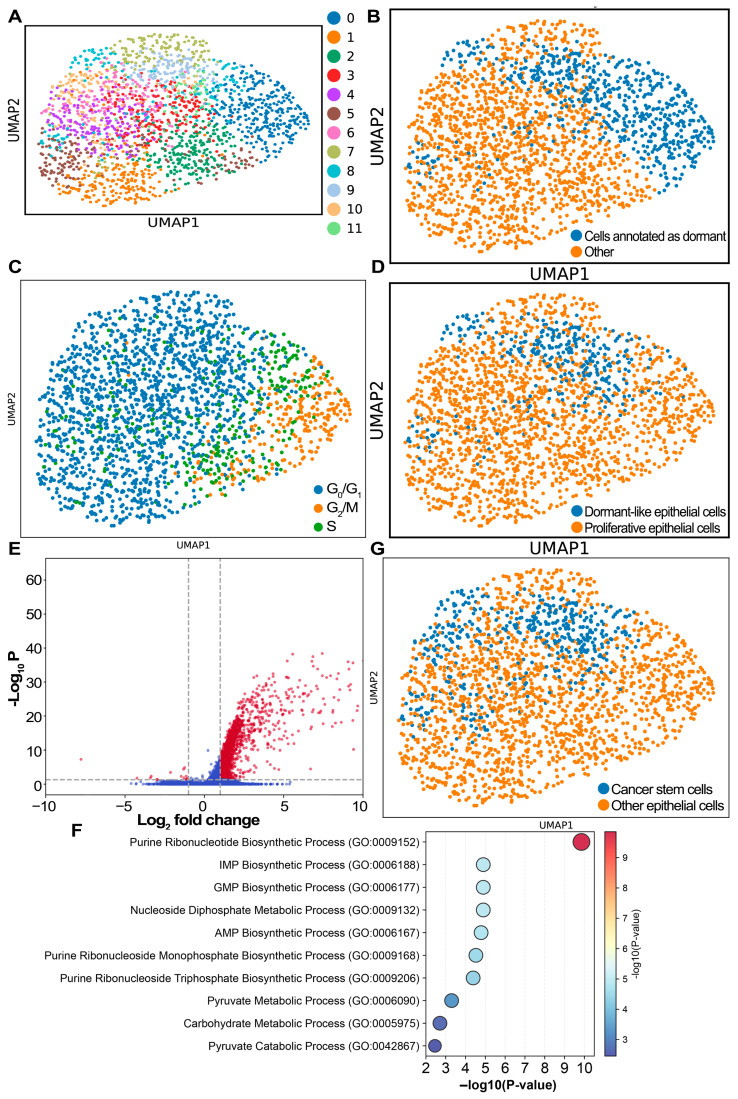
Dormant-like epithelial cells and gene signatures. (**A**) Epithelial cell clusters. (**B**) Dormant epithelial cell annotation. (**C**) Cell cycle analysis of epithelial cells. (**D**) Dormant-like epithelial cells. (**E**) Volcano plot for significantly differentially expressed genes, i.e., gene signatures, of dormant-like epithelial cells. (**F**) Enriched GO terms for gene signatures of dormant-like epithelial cells in CRC dormancy-related metabolic pathways. (**G**) Cancer stem cell annotation.

The epithelial cells were reclustered into 12 clusters (Figure 2A). Using dormancy marker genes, 4 clusters were annotated as dormant (Figure 2B). During cell cycle analysis, cells in these clusters were annotated as G_0_/G_1_ phase (Figure 2C). Thus, 292 cells were defined as dormant-like epithelial cells (Figure 2D). These dormant-like cells are a contributing factor to CRC metastasis.

The differential expression analysis of the dormant-like and proliferative epithelial cells yielded 2202 significantly differentially expressed genes (Figure 2E). Of these gene signatures of dormant-like epithelial cells, 14 genes were in dormancy-related metabolic pathways (Table S1). Thirteen of these genes were upregulated in dormant-like epithelial cells, suggesting a potential role in CRC dormancy.

Gene Ontology (GO) enrichment analysis was performed for these 14 genes, and 88 biological process (BP) terms were obtained (Figure 2F). Purine nucleobase biosynthetic processes were primarily involved. This enrichment suggested that epithelial cells modulated nucleotide availability to regulate their metabolic state during dormancy, which may in turn affect their immunosurveillance capacity [10]. These findings are consistent with the CRC dormancy-related metabolic pathways identified in our earlier analysis.

The core of cancer cell dormancy lies in cancer stem cells [11]. The epithelial cell clusters were further annotated as cancer stem cells and other epithelial cells according to cancer stem cell markers (Figure 2G). In cancer stem cells, 250 cells were dormant, while all the others were proliferative. Statistical comparison (Fisher’s exact test) confirmed that the proportion of dormant-like cells was significantly higher in cancer stem cells (250/460, 54.3%) than in non-stem epithelial cells (42/1581, 2.7%; *p* < 0.0001). This strong association between cancer stem cell identity and dormancy supports the validity of our re-annotation.

LGR5 is a well-established marker of colorectal cancer stem cells. Importantly, studies have shown that LGR5^+^ cancer stem cells exist in a dormant state marked by p27 expression, which allows them to persist through chemotherapy and drive recurrence [12]. Our re-annotation identified LGR5 expressed predominantly in the cancer stem cell clusters, consistent with their known dormant phenotype.

#### 2.2.3. Dormant-like Immune Cells and Gene Signatures

**Figure 3:**
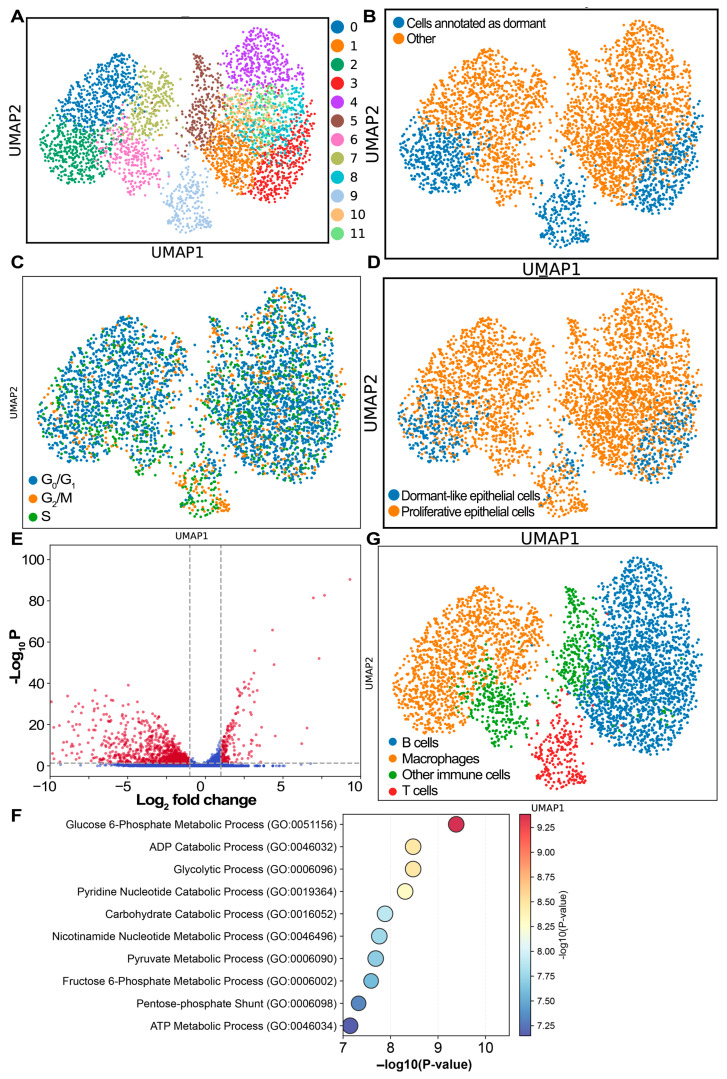
Dormant-like immune cells and gene signatures. (**A**) Immune cell clusters. (**B**) Dormant immune cell annotation. (**C**) Cell cycle analysis of immune cells. (**D**) Dormant-like immune cells. (**E**) Volcano plot for significantly differentially expressed genes, i.e., gene signatures, of dormant-like immune cells. (**F**) Enriched GO terms for gene signatures of dormant-like immune cells in CRC dormancy-related metabolic pathways. (**G**) Immune cell subtypes.

Clustering of immune cells yielded 12 cell clusters (Figure 3A). Using marker genes associated with dormancy, dormant cell clusters were annotated (Figure 3B). Cell cycle analysis further refined G_0_/G_1_ phase cells (Figure 3C). The intersection cells were final dormant-like immune cells, while the others were categorized as proliferative (Figure 3D). A total of 494 immune cells were dormant-like. They contributed to the decline of immune surveillance or the formation of an immunosuppressive environment, which might lead to cancer metastasis or recurrence.

Differential expression analysis of the dormant-like and proliferative immune cells obtained 1196 significantly differentially expressed genes (Figure 3E). The dormant-like immune cell gene signatures that were also in dormancy-related metabolic pathways were 13 genes (Table S1). These downregulated genes in dormant-like immune cells might contribute to CRC dormancy.

GO enrichment analysis was performed on these 13 genes, and 83 BP terms were acquired (Figure 3F). These genes were significantly enriched in glycolytic processes. This enrichment suggested that immune cells during dormancy might maintain energy homeostasis through enhanced glycolytic activity, providing necessary ATP and biosynthetic precursors while minimizing oxidative stress [1]. Such metabolic reprogramming could contribute to the establishment of an immunosuppressive microenvironment, potentially facilitating CRC metastasis.

The immune cell clusters were further annotated as different subtypes according to immune cell subtype markers (Figure 3G). B cells are the predominant immune cell type, followed by T cells and macrophages, consistent with the predicted cell types from CellTypist (Figure S8B). Among the 1858 B cells, 434 (23.4%) were classified as dormant-like; among 232 T cells, 60 (25.9%) were dormant-like. In contrast, no macrophages were found to be dormant (0%). Notably, no macrophages fulfilled the dormancy criteria, consistent with the absence of a quiescent cell cycle state in differentiated myeloid cells. These proportions are biologically reasonable and further support the validity of our annotation.

Among the B cell markers, *XBP1* is a transcription factor that drives plasma cell differentiation. Importantly, plasma cell differentiation is accompanied by cell cycle exit and cessation of proliferation [13]. *IL32* is overexpressed in CRC T cells and promotes metastasis and lymph node metastasis [14]. Metastasis is a consequence of dormant cell reactivation and dissemination. High IL32 expression likely reflects the reversal of dormancy, whereas low IL32 expression may be permissive for maintaining the dormant state.

### 2.3. CDAGs and Validation

#### 2.3.1. Preliminary Assessment of CDAGs

CDAGs were those that were both dormancy-related gene signatures of three main cell types from single-cell analysis and in CRC dormancy-related metabolic pathways acquired by the GMMPS algorithm. The 23 identified genes might play a regulatory role in the dormant state of tumor cells by modulating metabolic processes that affect energy supply or substance synthesis (Table S1).

Literature review confirmed that several CDAGs are directly implicated in CRC dormancy or dormancy-related processes. For example, ALDH1B1 regulates Wnt/β-catenin and PI3K/Akt pathways, linking stemness maintenance and dormancy awakening [15]. PKM alternative splicing affects chemoresistance and dormancy-proliferation transitions [16]. PRDX6 induces cell cycle arrest and ROS production, consistent with quiescence [17]. HK1, HK3, and PFKFB3 have been shown to influence CRC metabolism and dormancy [18,19]. Other CDAGs are associated with CRC progression, metastasis, or poor prognosis (e.g., *ALDH2*, *TKT*, *LDHB*, *AMPD2*, *IMPDH2*, *RRM2*, *ACP5*), providing orthogonal support for their biological relevance.

**Figure 4:**
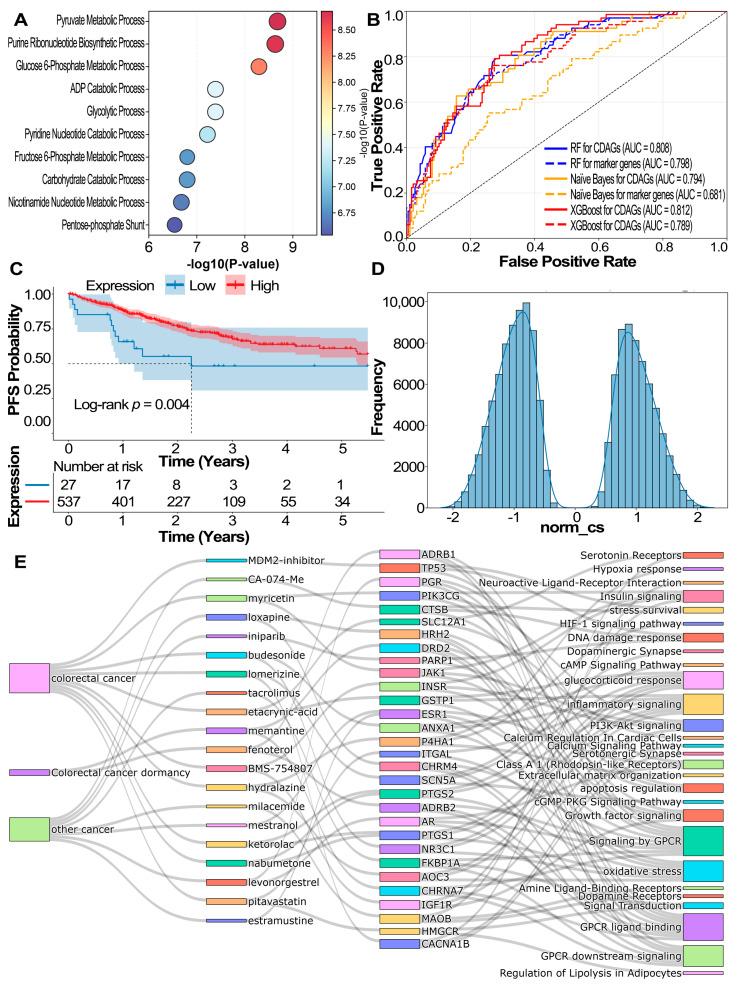
CRC dormancy-associated gene analysis and drug identification. (**A**) Enriched GO terms of CDAGs. (**B**) The ROC curve for five-fold cross-validation of machine-learning models for GSE41258 comparing CDAGs vs. dormancy marker genes. (**C**) Kaplan–Meier survival curves of CDAGs for TCGA COADREAD. (**D**) The norm_cs distribution for all drugs. (**E**) CRC dormancy-associated drugs and corresponding evidences.

GO enrichment analysis of all the CDAGs yielded 98 BP terms (Figure 4A). These genes were significantly enriched in Pyruvate Metabolic Process, Purine Ribonucleotide Biosynthetic Process, Glucose 6-Phosphate Metabolic Process, and Fructose 6-Phosphate Metabolic Process, suggesting that these biological processes might play important roles in CRC dormancy.

The clinical utility of CDAGs was further evaluated in large independent cohorts through metastasis classification and survival analysis (see Section 2.3.2, Section 2.3.3 and Section 2.3.4 for details).

#### 2.3.2. CDAGs Discriminate Metastatic from Non-Metastatic Samples

CDAGs were further validated in two independent external cohorts, GSE41258 and GSE41568, to assess their ability to distinguish metastatic from non-metastatic colorectal cancer samples. In both datasets, CDAGs consistently achieved numerically higher predictive performance than previously reported dormancy marker genes across multiple machine-learning models.

In GSE41258 (*n* = 253), CDAGs achieved higher performance in the RF model, with an AUC of 0.808 (95% confidence interval (CI): 0.749–0.864), compared to 0.798 (95% CI: 0.739–0.856) for marker genes. Bootstrap-based comparison showed ΔAUC did not reach statistical significance (ΔAUC = 0.010, 95% CI: −0.050 to 0.074, *p* = 0.372). Similar AUC improvements were observed with the Naïve Bayes model (CDAGs: 0.794 (95% CI: 0.731–0.852) vs. marker genes: 0.681 (95% CI: 0.609–0.750)) (ΔAUC = 0.114, 95% CI: 0.033–0.198, *p* = 0.008), and XGBoost (0.812 (95% CI: 0.756–0.866) vs. 0.789 (95% CI: 0.727–0.849)) (ΔAUC = 0.023, 95% CI: −0.036 to 0.082, *p* = 0.244), consistent with the RF findings (Figure 4B and Table S2). Decision curve analysis (DCA) showed that CDAGs yielded higher net benefit (NB) than the marker genes across a threshold probability range of approximately 0.25–0.35. Within the RF framework, CDAGs produced a net reclassification improvement (NRI) of 0.124 (95% CI: −0.155~0.412, *p* = 0.414) and an integrated discrimination improvement (IDI) of 0.022 (95% CI: −0.027~0.074, *p* = 0.408). In the XGBoost model, similar trends in AUC, NB, and reclassification metrics were observed, although the improvements were generally smaller and did not reach statistical significance (Figure S10A and Table S3). However, in the Naïve Bayes model, CDAGs yielded a statistically significant IDI of 0.187 (95% CI: 0.039–0.325, *p* = 0.010), whereas the NRI was not, indicating improved discrimination of continuous metastatic risk estimates despite the absence of significant NRI improvement.

In GSE41568 (*n* = 133), the RF model with CDAGs achieved an AUC of 0.864 (95% CI: 0.798–0.924), compared with 0.788 (95% CI: 0.702–0.874) for the marker genes. Bootstrap-based comparison showed a ΔAUC of 0.076 (95% CI: 0.002–0.155, *p* = 0.024). The Naïve Bayes model (0.795 (95% CI 0.708–0.880) vs. 0.740 (95% CI 0.644–0.826)) (ΔAUC = 0.058, 95% CI: −0.065~0.175, *p* = 0.183)) and XGBoost (0.871 (95% CI: 0.807–0.929) vs. 0.786 (95% CI: 0.698–0.871)) (ΔAUC = 0.087, 95% CI: −0.002 to 0.175, *p* = 0.031) showed analogous AUC patterns (Figure S11A). DCA demonstrated that CDAGs provided higher NB over a broad range of thresholds. In the RF framework, CDAGs yielded an NRI of 0.491 (95% CI: 0.094–0.838, *p* = 0.012) and an IDI of 0.073 (95% CI: 0.008–0.134, *p* = 0.024). Naïve Bayes and XGBoost also demonstrated significant NRI improvement, whereas the IDI did not reach significance (Figure S10B and Table S3). Overall, CDAGs showed higher AUC values and consistently higher net benefit across a broad range of thresholds across all models in the RF model, suggesting a trend that warrants further investigation.

To preliminarily assess whether these 23 CDAGs are associated with the dormant state, we evaluated their classification performance in an independent small cohort (GSE193248, *n* = 15) by establishing a Random Forest (RF) model. The Area Under the Curve (AUC) value of a five-fold cross-validation was 0.907, suggesting a potential association with dormancy. Leave-one-out cross-validation (LOOCV) yielded an AUC of 0.889, which should be interpreted with caution and considered exploratory only, as the sample size is too small to provide stable performance estimates.

While M1/M0 status is a clinical proxy rather than a direct measure of dormancy, the consistent discriminative performance of CDAGs across both classification cohorts suggests that these genes capture biologically relevant features of metastatic competence.

#### 2.3.3. Prognostic Value of CDAGs

To avoid potential overfitting from selecting a cut-off on the same dataset used for testing, we first determined an optimal expression cut-off for stratifying high- and low-risk patients using the independent GSE41258 cohort (*n* = 217) with recurrence-free survival (RFS) as the endpoint. The cut-off was obtained using the maximally selected rank statistic (surv_cutpoint function in the survminer R package, version 0.5.2). In this cohort, high expression of CDAGs was significantly associated with better RFS (*p* = 0.005) (Figure S11B), while dormancy marker genes failed to reach statistical significance (*p* = 0.068). To evaluate whether the prognostic value of the CDAGs is independent of other variables, we performed a multivariate Cox regression adjusting for age, sex, and American Joint Committee on Cancer (AJCC) stage. These genes maintained independent prognostic value (hazard ratio (HR) = 0.74, 95% CI: 0.55–0.98, *p* = 0.037).

To evaluate the potential clinical utility of our CDAGs for prognostic prediction, we performed DCA comparing CDAGs with marker genes. DCA showed that over the 15–30% threshold range, CDAGs demonstrated numerically higher net benefit compared with marker genes (mean NB: 0.0115 vs. 0.0066). We further calculated the NRI and IDI to assess whether our CDAGs improved risk classification. The NRI was −0.0108 (95% CI: −0.279 to 0.260, *p* = 0.932), and the IDI was 0.0805 (95% CI: −0.050 to 0.207, *p* = 0.218), indicating no statistically significant improvement in risk reclassification (Figure S10D).

The same cut-off (derived from GSE41258) was then applied to The Cancer Genome Atlas (TCGA) Colon and Rectal Cancer (COADREAD) cohort (*n* = 564) for progression-free survival (PFS) analysis. Survival analysis (truncated at 2000 days) showed that high expression of CDAGs was significantly related to Progression-Free Survival (PFS) (log-rank *p* = 0.004). The Kaplan–Meier curve with the number at risk at 0, 1, 2, 3, 4, and 5 years is shown in Figure 4C. In contrast, dormancy marker genes failed to reach statistical significance in stratifying patients based on PFS in the same cohort (*p* = 0.698). Multivariate Cox regression adjusting for age, sex, AJCC stage and molecular subtype confirmed their independent prognostic value (HR = 0.40, 95% CI: 0.25–0.83, *p* = 0.009).

CDAGs showed higher net benefit (mean NB: 0.0144 vs. −0.0066) over the 15–30% threshold range, with ΔNB ranging from 0.008 to 0.027 across all thresholds (Figure S10C). The NRI was −0.0176 (95% CI: −0.178 to 0.152, *p* = 0.89), and the IDI was 0.0065 (95% CI: −0.020 to 0.034, *p* = 0.65), consistent with the training set findings.

These results demonstrated that the group with high expression of CDAGs showed significantly better PFS/RFS in both the TCGA COADREAD data and the independent GSE41258 cohort. This suggested that these CDAGs might help identify individuals whose tumor cells were in a stable dormant-like state and who had a better clinical prognosis. It also suggested that maintaining tumor dormancy might be a potential strategy for preventing and treating CRC metastasis.

#### 2.3.4. CRC Dormancy-Associated Drugs

In this study, the Connectivity Map (CMap) database was utilized to identify CRC dormancy-associated drugs (see Section 4 for details). A total of 22 CDAGs, including *ALDH1B1*, *ALDH2*, *TKT*, *AK2*, *LDHB*, *AMPD2*, *APRT*, *ATIC*, *PKM*, *IMPDH2*, *NME1*, *PRDX6*, *TXN*, *ALDH3B1*, *ACP5*, *FBP1*, *BLVRB*, *HK1*, *PGD*, *HK3*, *PFKFB3*, and *TALDO1*, were used as the upregulated gene input for CMap analysis, whereas no downregulated gene set was included in the final query. We analyzed the distribution of normalized connectivity scores (norm_cs) for all compound perturbations (Figure 4D). Interestingly, the distribution exhibited a bimodal pattern, with prominent peaks located at both the positive and negative ends of the connectivity spectrum, whereas relatively few compounds were concentrated near the center of the distribution. This pattern suggests that many compounds produced either strong transcriptional concordance or strong transcriptional opposition relative to the CDAG expression signature, rather than weak or neutral transcriptional effects. Compounds with strongly positive connectivity scores were considered of particular interest in the present study, as they may potentially induce or maintain dormancy-associated transcriptional programs in CRC. Conversely, compounds with strongly negative connectivity scores may represent agents capable of reversing dormancy-related molecular states.

A total of 20 drugs with transcriptional signatures most similar to the CDAG profile were identified (Figure 4E). Among the 20 identified drugs, 12 have been previously associated with CRC in the literature. Of these, ten compounds have direct experimental or clinical evidence of anti-proliferative or anti-metastatic activity. For example, memantine has demonstrated potential anti-cancer effects as an adjunctive therapy in metastatic colon cancer [20]. Pitavastatin has been reported to overcome chemoresistance in metastatic CRC under high-glucose conditions, supporting a role for metabolic regulation in CRC progression [21]. The flavonoid myricetin has also shown anti-colorectal cancer activity in vitro and in silico [22]. Lomerizine 2HCl has been shown to inhibit CRC cell proliferation and induce protective autophagy via the PI3K/Akt/mTOR signaling pathway [23]. BMS-754807, an IGF-1R/IR inhibitor, has demonstrated anti-proliferative effects in colon cancer cell models [24].

Additional compounds have been implicated in other cancer types. These drugs might act on dormancy-related metabolic pathways and contribute to CRC dormancy. Pathway enrichment analysis on the targets of these drugs revealed that the predicted drug targets were significantly enriched in several signaling pathways closely related to tumor dormancy. Among the most significantly enriched pathways were GPCR ligand binding, signaling by GPCR [25], neuroactive ligand–receptor interaction, serotonergic synapse, dopaminergic synapse, adrenoceptor signaling, muscarinic acetylcholine receptor signaling, and calcium signaling pathways [26]. These pathways were primarily driven by multiple serotonin receptors (HTR family), dopamine receptors (DRD family), adrenergic receptors (ADRA/ADRB family), muscarinic acetylcholine receptors (CHRM family), and other neurotransmitter-associated receptors. Emerging evidence indicates that neurotransmitter signaling and GPCR-mediated communication can regulate tumor cell plasticity, stress tolerance, immune evasion, and dormancy maintenance within the metastatic niche [27]. Furthermore, calcium signaling and cAMP-dependent pathways have been implicated in reversible cell-cycle arrest and adaptation to nutrient or hypoxic stress [28]. Moreover, pathways involved in metabolic regulation and growth signaling were prominently represented, including the PI3K-Akt signaling pathway, AMPK signaling pathway, and mTOR signaling pathway. These pathways are well known to regulate the balance between cellular proliferation and quiescence in cancer cells [29]. In addition, enrichment of the HIF-1 signaling pathway and insulin signaling pathway suggests that hypoxia adaptation and metabolic stress responses may also contribute to the maintenance of dormant tumor cells [30]. Previous studies have demonstrated that suppression of PI3K-AKT-mTOR signaling together with activation of AMPK-mediated energy stress responses can promote entry of tumor cells into a dormant state. These findings suggested that the CRC dormancy-associated drugs might influence colorectal cancer progression by modulating signaling networks associated with tumor cell dormancy, providing a mechanistic rationale for their potential as dormancy-promoting agents.

## 3. Discussion

In this paper, we comprehensively examined CRC dormancy by integrating bulk and single-cell analysis. Twenty-three CDAGs that were both in dormancy-related metabolic pathways and gene signatures in dormant-like epithelial cells, cancer stem cells and immune cells were identified. Our CDAGs shared no overlapping genes with the previously published dormancy marker genes. Pathway enrichment analysis of marker genes revealed predominant involvement of immune-related processes, including T cell receptor signaling, PD-1/PD-L1 pathway, and antigen processing and presentation. In contrast, our CDAGs were enriched in metabolic pathways such as fatty acid oxidation, glycolysis, and purine metabolism. These distinct enrichment patterns suggested that the two gene sets might capture complementary aspects of tumor dormancy: the marker genes primarily reflected immune microenvironment interactions, while our genes participated in intrinsic tumor cell metabolic reprogramming associated with dormancy.

This study specifically characterized the functional impact of genes in metabolic pathways on CRC dormancy. Metabolites from these CRC dormancy-related pathways also contributed to CRC dormancy regulation. Acetyl-CoA, a central metabolite in Pyruvate metabolism, serves as the fundamental precursor for both energy substrates (fatty acids and ketone bodies) and bioactive compounds (cholesterol and its derivatives). Excess acetyl-CoA from fatty acid oxidation is converted to cytosolic acetyl-CoA by ATP-citrate lyase, which activates Nanog transcription and drives CRC into dormancy [31]. Cholesterol, a central metabolite in Cholesterol biosynthesis 1 (Bloch pathway), plays a critical role in mediating immunotherapy resistance in CRC. Elevated cholesterol biosynthesis contributes to distal recurrence in CRC. The cholesterol synthesis pathway represents a potential diagnostic and therapeutic target for controlling CRC metastasis [32].

Single-cell analysis has become a standard methodology in cancer research for characterizing tumor stem cells, drug-resistant populations, and metastatic subclones. However, its application to cancer dormancy studies, especially in CRC, remains limited. This study implemented single-cell analysis to investigate dormancy mechanisms and characterize distinct dormant-like cells. Within the immune microenvironment, epithelial cells and immune cells demonstrate functional interactions. The proliferative and dormant states of these cellular populations are regulated through dynamic equilibrium between cell cycle activators and repressors [33]. Consequently, our single-cell analysis incorporated epithelial and immune cells to enable comprehensive characterization and mechanistic investigation. Our single-cell analysis integrated samples from multiple patients, which might introduce batch effects and inter-patient heterogeneity. While we used standard integration methods to align the data, some residual variability may persist. Nevertheless, the observation of dormant-like cells across individual samples and the robust validation of CDAGs in large external cohorts suggest that the main conclusions are not driven by batch effects or sample variability. To further characterize the dormancy state, we applied CytoTRACE2 to the scRNA-seq data. CytoTRACE2 scores were compared between dormant-like and proliferative cells using the Wilcoxon rank-sum test with Bonferroni correction. Epithelial dormant-like cells showed significantly higher CytoTRACE2 scores than proliferative epithelial cells (*p* < 0.001), indicating a more stem-like state. In contrast, dormant-like immune cells exhibited significantly lower CytoTRACE2 scores (*p* < 0.001), suggesting a more differentiated phenotype (Figure S9). These distinct patterns are consistent with cell-type-specific dormancy programs.

The integration of single-cell transcriptomics with metabolomics has become a powerful approach for elucidating cellular heterogeneity, functional states, and microenvironmental interactions. These complementary methodologies synergistically uncover fundamental molecular mechanisms underlying biological processes.

To further interpret the contribution of individual genes to the predictive model, SHapley Additive exPlanations (SHAP) analysis was performed to quantify the importance of each feature in RF classifiers for two independent CRC cohorts (GSE41258 and GSE41568). In both cohorts, genes involved in nucleotide metabolism (*IMPDH2*, RRM2), glycolysis (*HK3*, *LDHB*), gluconeogenesis (*FBP1*), redox homeostasis (*PGD*, *TXN*), and energy transfer (*AK2*, *NME1*) consistently emerged as top contributors, demonstrating cross-dataset stability (Figure S12A,B). *RRM2* and *IMPDH2* are key regulators of nucleotide synthesis and DNA replication, supporting proliferative recovery and therapy resistance under stress conditions [34,35]. *PGD* and *TXN* contribute to redox homeostasis by regulating pentose phosphate pathway activity and antioxidant defense, thereby facilitating adaptation to oxidative stress [36,37]. *HK3* and *LDHB* participate in glycolytic and lactate metabolic pathways that enable metabolic flexibility in nutrient-limited microenvironments [18,38], whereas *FBP1* has been implicated in modulating glycolytic dependency and metastatic plasticity [39]. In addition, *NME1* and *AK2* are involved in nucleotide homeostasis and cellular energy transfer, further supporting metabolic adaptation during tumor progression [40,41]. These findings not only validated the reliability of the predictive models, but also identified a panel of reproducible and biologically plausible metastasis-associated candidate biomarkers.

To further explore the clinical context in which the signature is most informative, we performed stratified analyses based on tumor stage and chemotherapy status. Stratified analyses revealed stage-dependent prognostic patterns. CDAGs showed strong significance in late-stage patients (Stage III–IV) in both GSE41258 (*p* = 0.001) and TCGA COADREAD (*p* < 0.001). In TCGA early-stage patients (Stage I–II), CDAGs did not reach significance (*p* = 0.426), likely due to limited events in this favorable-prognosis subgroup; GSE41258 early-stage patients were not analyzed due to insufficient events (events = 7). Among TCGA patients not receiving radiation (*p* = 0.010), with lymph node metastasis (N2: HR = 0.36, *p* = 0.0164), and with distant metastasis (M1: HR = 0.35, *p* = 0.0360), CDAGs remained significant. Notably, within the CIN molecular subtype—the predominant CRC subtype (55.9% of the cohort)—CDAGs remained significantly prognostic (HR = 0.43, 95% CI: 0.21–0.90, *p* = 0.0212), suggesting specific utility in this biologically defined context. These findings suggest potential stage-modulated prognostic utility.

There are some limitations in this study. The bulk dataset used in this study was initially derived from CRC cell lines, which may not fully recapitulate the complex microenvironmental cues that influence tumor dormancy in patients. While we observed consistent correlations with dormancy markers and clinical outcomes in our patient-derived single-cell datasets, suggesting a degree of conserved metabolic features, this approach assumes a level of biological equivalence that warrants further investigation. Future studies utilizing in vivo models or patient-derived organoids are necessary to functionally validate the metabolic dependencies identified here and to determine their role in maintaining dormancy within a native tumor microenvironment. The GMMPS algorithm used in this study only obtained pathway activity from bulk gene expression data without considering the temporal dynamics of metabolite concentrations. This might limit the mechanistic interpretation of causality. Additionally, the currently available data on the dormancy of gastrointestinal cancers is limited. Although independent datasets were used to validate the classification performance of CDAGs, they rely on clinical metastasis status (M1/M0) rather than direct assessment of dormancy. Further validation in cohorts with direct dormancy markers is needed to confirm these findings. The transcriptomic analyses presented here do not establish causal relationships between the identified genes and the induction or maintenance of dormancy. Similarly, while the computational predictions presented in this study provide a valuable resource for identifying potential dormancy-associated drugs, functional validation through in vitro and in vivo experiments is necessary. While our literature review identified several predicted drugs with established anti-metastatic activity, future studies are needed to formally test whether these compounds indeed induce or maintain a dormant phenotype in CRC cells.

As a primarily computational study with limited experimental validation, our findings are hypothesis-generating and require functional confirmation. To facilitate translation of computational findings into clinical practice, the following stepwise validation plan should be pursued in future studies. The top CDAGs including *HK3*, *ALDOB*, and *RRM2* should be tested in CRC cell lines such as HCT116 and HT29 using established dormancy models including serum starvation, three-dimensional culture, and label-retention assays. Readouts would include Ki-67 index, EdU incorporation, cell cycle distribution by fluorescence-activated cell sorting, and dormancy marker expression including p27, *DEC2*, and *NR2F1*. For in vivo validation, orthotopic or liver metastasis models in immunocompromised mice with CRISPR-mediated perturbation of candidate CDAGs would assess metastatic burden, dormant lesion frequency by histology, and time-to-progression. These experiments would clarify whether CDAGs actively regulate metastatic latency, survival, or reactivation. A retrospective cohort study using formalin-fixed paraffin-embedded primary tumors from two hundred to three hundred stage II-III CRC patients with known recurrence outcomes should distinguish synchronous metastasis, metachronous relapse, and long-term metastasis-free survival to define the relationship between dormancy-associated transcriptional programs and metastatic progression. CDAG expression would be measured by RT-qPCR or RNA sequencing with recurrence-free survival as the primary endpoint. A subsequent prospective pilot in fifty to one hundred high-risk stage II patients would measure CDAGs at diagnosis and monitor circulating tumor DNA as a surrogate for minimal residual disease. From a translational perspective, these signatures may ultimately contribute to postoperative risk stratification, surveillance optimization, and liquid biopsy-based monitoring. Dormancy-associated drugs identified by CMap should be screened in patient-derived organoids from CDAG-high versus CDAG-low tumors, followed by a biomarker-stratified randomized clinical trial in the adjuvant setting to test whether dormancy-maintenance therapy improves outcomes compared to standard observation.

## 4. Materials and Methods

**Figure 5:**
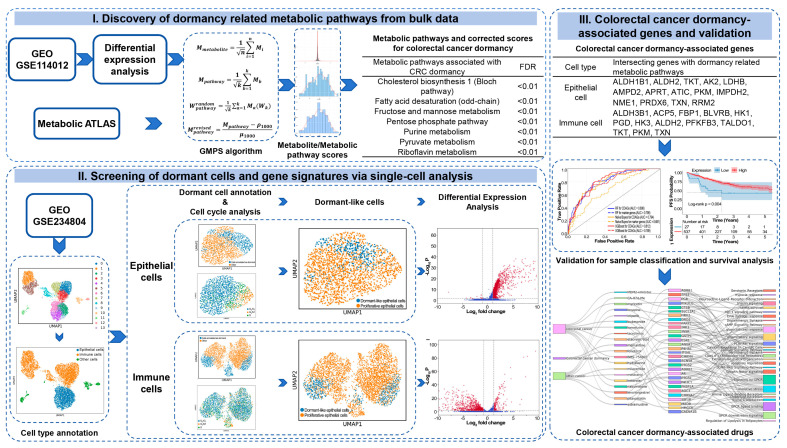
Flow chart. The analysis consists of three steps: (**I**) discovery of dormancy-related metabolic pathways based on the GMMPS algorithm, (**II**) screening of dormant-like cells and gene signatures by single-cell analysis, and (**III**) identification of CDAGs and drugs.

The overall analysis workflow is illustrated in Figure 5.

### 4.1. Data

The expression data were obtained from the Gene Expression Omnibus (GEO) database [42] (Table 1). The bulk dataset GSE114012 included 48 samples with four replicates of both dormant and proliferative states across six CRC cell lines. Analysis of gene expression differences between dormant and proliferative samples revealed 645 differentially expressed genes (see Section 4.5 for statistical details).

The dataset GSE234804 comprised single-cell RNA sequencing data from 3 primary CRC, 6 liver metastasis and 4 peritoneal cancer samples. To identify dormant-like cells and gene signatures, data for primary CRC and liver metastasis samples were utilized.

Further assessment and validation were performed using multiple independent datasets. GSE193248 (*n* = 15, 9 metastatic and 6 micrometastatic (dormant)) was used for exploratory assessment of CDAG association with the dormant state. Two independent external cohorts, GSE41258 (*n* = 253, 67 metastatic and 186 non-metastatic) and GSE41568 (*n* = 133, 94 metastatic and 39 non-metastatic), were used to evaluate the ability of CDAGs to distinguish metastatic from non-metastatic samples with machine-learning models. To assess the clinical relevance of CDAGs in predicting dormancy-related outcomes, PFS and RFS were used as endpoints. Accordingly, datasets with PFS or RFS information were used for survival analysis. TCGA COADREAD data with PFS were obtained from [48] (*n* = 564). PFS was defined as time from diagnosis to first progression or death, consistent with the TCGA protocol. The clinical characteristics of the patients from TCGA COADREAD are provided in Table S4. GSE41258 with RFS (total *n* = 253, of which 217 had RFS information) was also used for external validation of prognostic value. RFS was defined as the time from diagnosis to the first documented recurrence.

The Metabolic ATLAS database (3.4) provides open-source genome-scale metabolic models (GEM) with metabolic pathways, metabolites and genes [49]. A total of 6239 metabolites were obtained from the database, along with 137 metabolic pathways from the Human-GEM for differentially expressed genes from the bulk dataset (862,856 metabolite-gene pairs and 10,471 metabolite–metabolic pathway pairs).

### 4.2. Discovery of Dormancy-Related Metabolic Pathways Based on the GMMPS Algorithm

In this paper, we developed a novel algorithm, GMMPS, to identify dormancy-related metabolic pathways. The algorithm was designed as an integrated framework that links gene-level differential expression to metabolite-level and pathway-level scores using prior knowledge from Metabolic ATLAS, while preserving the directionality of gene expression changes throughout the aggregation steps.

Based on the relationships between differential genes from the bulk dataset and metabolites derived from the Metabolic ATLAS database, each metabolite was assigned a score indicating the potential effect of perturbations in gene expression on the metabolite (Equation (1)):

(1) $$M_{metabolite}=\frac{1}{\sqrt{n}}\sum_{i=1}^{n}M_{i},$$

Here, $M_{i}$ is the Z score of gene i obtained from the corresponding *p* value by using the inverse normal cumulative distribution, and n is the number of related genes of the metabolite.

Each metabolic pathway was then assigned a score based on the relationships between metabolites and metabolic pathways (Equation (2)):

(2) $$M_{pathway}=\frac{1}{\sqrt{k}}\sum_{b=1}^{k}M_{b},$$

Here, $M_{b}$ is the Z score of metabolite b obtained by the inverse normal cumulative distribution of the score $M_{metabolite}$ of the corresponding metabolite, and k is the number of metabolites associated with this pathway.

In order to determine the significance of the pathway score, the same number of metabolites was randomly selected for each metabolic pathway to calculate a random score (Equation (3)), and the process was repeated 1000 times.

(3) $$W_{pathway}^{random}=\frac{1}{\sqrt{k}}\sum_{a=1}^{k}M_{b}\left(W_{k}\right),$$

Here, k is the number of metabolites contained by the metabolic pathway. $M_{b}\left(W_{k}\right)$ represents the sum of Z scores of k metabolites randomly selected from $M_{b}$.

The metabolic pathway score was then corrected (Equation (4)):

(4) $$M_{pathway}^{corrected}=\frac{M_{pathway}-\rho_{1000}}{\mu_{1000}},$$

Here, $\rho_{1000}$ represents the average value of 1000 random pathway scores, and $\mu_{1000}$ represents the standard deviation of them.

Among the metabolic pathways that remained statistically significant (FDR < 0.05), the top 5% with the largest $\left|M_{pathway}^{corrected}\right|$ were determined as CRC dormancy-related metabolic pathways (see Section 4.5 for permutation test details).

### 4.3. Screening of Dormant-like Cells and Gene Signatures by Single-Cell Analysis

Single-cell analysis was performed using Python 3.13 (Python Software Foundation, Wilmington, DE, USA). Each sample was filtered (200~5000 genes per cell, ≥3 cells per gene) and merged. Doublets were removed with Scrublet (batch_key = sample). Cells with >15% mitochondrial reads were removed. Data were normalized (median-total scaling + log1p). Highly variable genes (top 2000) were selected. Principal Component Analysis (PCA) and Uniform Manifold Approximation and Projection (UMAP) were performed for dimensionality reduction and visualization. Batch effects were corrected using scVI-tools. A neighborhood graph was constructed and clustering was performed using the Leiden algorithm with a resolution of 1.0, after exploring a range of resolutions (0.1–1.0). 

#### 4.3.1. Cell Type Annotation

Cell type annotation was performed based on classical marker genes (Figure S7). Cells corresponding to epithelial and immune cells were isolated for subsequent reclustering and detailed characterization.

For immune cells, further subtype annotations were performed, which included B cells, T cells, and macrophages.

#### 4.3.2. Screening of Dormant-like Cells and Gene Signatures

Based on the dormancy mechanisms, this study identified and characterized dormant-like cells by focusing on tumor microenvironmental regulation and cell-cycle arrest.

Cells were annotated as dormant according to dormancy marker genes compiled from a targeted literature review of PubMed, focusing on studies that explicitly identified or validated dormancy-associated markers (Table S5). For epithelial cells, only dormancy marker genes detected in ≥40% of cells were considered. For immune cells, a lower threshold of 20% was applied because the curated dormancy markers are primarily epithelial-derived genes that are less abundantly expressed in immune populations. Cells with three or more such genes were classified as dormant.

Cell cycle phase was determined using the scib.preprocessing.score_cell_cycle function.

The final dormant-like cells were those that were annotated as dormant based on dormancy marker genes and were simultaneously in the G_0_/G_1_ phase, while the rest were classified as proliferative cells. Subsequently, differential expression analyses were performed on the dormant and proliferative cells to identify significantly differentially expressed genes meeting the statistical criteria described in Section 4.5. These genes were dormancy-related gene signatures in each cell type. The Gene Ontology (GO) enrichment of these gene signatures was performed (see Section 4.5 for statistical thresholds).

### 4.4. Identification and Validation of CDAGs

#### 4.4.1. Identification and Preliminary Characterization of CDAGs

Dormancy-related gene signatures from single-cell analysis that were in CRC dormancy-related metabolic pathways acquired by the GMMPS algorithm were designated as CDAGs. GO enrichment analysis was then performed to interpret their biological functions.

To preliminarily evaluate whether the identified 23 CDAGs are associated with dormancy, we performed a classification analysis using an independent small cohort GSE193248 (*n* = 15). This dataset contained nine metastatic and six micrometastatic (dormant) CRC samples. A RF classifier was trained using the expression values of CDAGs as features and sample status (dormant vs. metastatic) as the binary outcome. Hyperparameter tuning was performed using GridSearchCV, with the optimal parameters set to n_estimators = 100 and default settings otherwise. To maximize the use of limited data, we employed five-fold cross-validation, where each fold used 12 samples for training and 3 for validation. Model performance was assessed using AUC values of Receiver Operating Characteristic (ROC) curves.

To assess whether CDAGs could predict metastasis and recurrence, we performed classification and survival analyses in larger cohorts as described below.

#### 4.4.2. Classification Analysis of CDAGs in Large Cohorts

To evaluate whether the CDAGs could distinguish metastatic from other samples, we performed sample classification in two independent datasets, GSE41258 (*n* = 253) and GSE41568 (*n* = 133), respectively. In these two cohorts, RF, Naïve Bayes and XGBoost classifiers were constructed based on the expression values of the CDAGs as features, with sample status (metastatic or not) as the binary outcome. Hyperparameters for each classifier were set as follows without additional tuning: For RF, n_estimators = 300, class_weight = ‘balanced’, max_depth = None; for Naïve Bayes, feature standardization (StandardScaler) followed by a GaussianNB classifier with default parameters; for XGBoost, n_estimators = 200, eval_metric = ‘logloss’, learning_rate = 0.3, max_depth = 6. For comparison, we also applied the same classifiers to dormancy marker genes we collected. Five-fold stratified cross-validation was applied to preserve the original proportion of metastatic and non-metastatic samples in each fold. Predictive performance was primarily evaluated using ROC curves and AUC values.

DCA was performed to assess the clinical utility of the CDAGs for classifying metastatic samples. Predicted probabilities for each sample were obtained from models trained on the same cohort using five-fold cross-validation to avoid overfitting. NB was calculated across threshold probabilities as:

(5) $${NB}_{cla}=\frac{{TP}_{cla}}{n_{cla}}-\frac{{FP}_{cla}}{n_{cla}}\times \frac{{pt}_{cla}}{1-{pt}_{cla}},$$

where ${TP}_{cla}$ is the number of samples correctly classified as metastatic (true positives), ${FP}_{cla}$ is the number of non-metastatic samples incorrectly classified as metastatic (false positives), ${pt}_{cla}$ is the threshold probability, and $n_{cla}$ is the total number of samples. NB curves were generated for both the CDAGs and existing dormancy marker genes we collected, as well as the default strategies of “classify all as metastatic” and “classify none as metastatic”. Bootstrap resampling (1000 iterations) was performed to assess the statistical significance of the observed difference in NB between CDAGs and marker genes. The 95% confidence intervals for the NB difference were derived from the 2.5th and 97.5th percentiles of the bootstrap distribution.

To quantify the incremental predictive value of CDAGs over existing dormancy marker genes, NRI and IDI were calculated for the classification of metastatic samples. Risk categories were defined based on tertiles of the predicted probabilities from the marker-gene model. NRI was calculated such that for metastatic samples, an increase in predicted probability from the marker-gene model to the CDAG model was considered correct upward reclassification; for non-metastatic samples, a decrease was considered correct downward reclassification. The NRI was then computed as the sum of the proportion of metastatic samples correctly reclassified upward and the proportion of non-metastatic samples correctly reclassified downward. Positive NRI values indicate improved performance of the CDAG model. IDI was calculated as the difference between the average predicted risk improvement for metastatic samples and that for non-metastatic samples when moving from the marker-gene model to the CDAG model. Positive values favor the CDAG model. Bootstrap resampling with 1000 iterations was performed to derive 95% confidence intervals and *p*-values.

#### 4.4.3. Survival Analysis of CDAGs in Large Cohorts

To evaluate the prognostic value of the CDAGs, we performed survival analysis using GSE41258 (*n* = 217) with RFS as the endpoint and TCGA COADREAD data (*n* = 564) with available PFS information, respectively. For each cohort independently, the signature score was calculated as follows: first, expression values of each of the 23 genes were Z-score normalized across all samples; second, the normalized values were averaged to obtain a raw signature score; third, the raw scores were further Z-score normalized to obtain the final signature score.

The optimal cut-off for defining high- and low-expression groups was determined in the GSE41258 cohort using the surv_cutpoint function in the survminer R package. This cut-off value was then applied to the TCGA COADREAD cohort for survival analysis. Kaplan–Meier curves were generated using the survival package, and survival differences were assessed by the log-rank test. To avoid unstable tail estimates of TCGA COADREAD data, follow-up was truncated at 2000 days (approximately 5.5 years), after which fewer than 5% of patients remained at risk. The number of patients at risk was reported at 0, 1, 2, 3, 4, and 5 years. Multivariate Cox proportional hazards models were used to evaluate the independent prognostic value of the CDAGs after adjusting for age (continuous), sex (binary), and AJCC stage as a categorical variable with Stage I as the reference category. Hazard ratios (HRs) with 95% confidence intervals were calculated from the model coefficients. For GSE41258, the entire follow-up period was used because more than 10% of patients remained at risk at the maximum follow-up time.

DCA was performed to assess the clinical utility of the CDAGs for predicting 3-year PFS/RFS. Predicted risk of PFS/RFS event at 3 years was derived from a Cox proportional hazards model including the signature expression level. NB was calculated as:

(6) $${NB}_{sur}=\frac{{TP}_{sur}}{n_{sur}}-\frac{{FP}_{sur}}{n_{sur}}\times \frac{{pt}_{sur}}{1-{pt}_{sur}},$$

where ${TP}_{sur}$ is the number of patients predicted to be at high risk who experienced the PFS/RFS event within 3 years, ${FP}_{sur}$ is the number predicted to be at high risk who did not experience the event within 3 years, ${pt}_{sur}$ is the threshold probability, and $n_{sur}$ is the total number of patients. NB curves were generated for both the CDAGs and existing dormancy marker genes, as well as the default strategies of “treat all” and “treat none”. Bootstrap resampling was performed to assess the statistical significance of the observed difference in NB. With 1000 iterations, a bootstrap sample of the same size was drawn with replacement, and NB at each threshold was recalculated for both gene sets. The 2.5th and 97.5th percentiles of the bootstrap estimates were used as 95% confidence intervals for NB. The difference in NB was computed per iteration, and its 95% confidence interval was derived. A difference was considered statistically meaningful if the confidence interval did not include zero.

NRI and IDI were calculated according to the method described in [50] to evaluate whether the CDAGs improved risk classification compared with the marker genes at 3 years. Risk categories were defined as low, intermediate, and high based on the 33rd and 67th percentiles of the risk scores from the marker-gene model. NRI was computed as the sum of the proportion of events correctly reclassified upward and nonevents correctly reclassified downward. IDI was computed as the difference in improvement in average risk scores between event and non-event groups. Bootstrap resampling with 1000 iterations was performed to derive 95% confidence intervals and *p*-values.

#### 4.4.4. Screening for Dormancy-Associated Drugs

The CMap database was employed to acquire dormancy-associated drugs with CDAGs as input. Results containing missing values in either moa (mechanism of action) or target_name (molecular target of pharmacological compounds) were removed. Compounds were ranked by their normalized connectivity score (norm_cs) and the top 20 compounds with the highest positive scores were selected for further analysis, excluding those with Broad Institute (BRD) prefix identifiers (which are non-specific chemical probes lacking well-defined mechanisms of action). This rank-based approach avoids an arbitrary fixed threshold. The pathway enrichment analysis for targets of these drugs was performed (see Section 4.5 for statistical thresholds).

### 4.5. Statistical Analysis

Statistical analyses were mainly performed using Python and R 4.2.1 (R Foundation for Statistical Computing, Vienna, Austria).

Differential expression between dormant and proliferative samples was analyzed using the DESeq2 package. Genes with a Benjamini–Hochberg adjusted *p*-value (FDR) < 0.05 and |log_2_FC| ≥ 1 were considered significant.

To assess the statistical significance of the corrected pathway scores, we constructed a null distribution for $M_{pathway}^{corrected}$ based on the permutation procedure. For each of the 1000 random iterations, a random pathway score was generated, and a corresponding ‘random corrected score’ was calculated using the same mean and standard deviation derived from the full set of random scores. This yielded a null distribution of 1000 random corrected scores. The empirical *p* value for a given pathway was calculated as the proportion of random corrected scores with absolute value greater than or equal to the absolute value of the observed corrected score. The resulting *p* values were then adjusted for multiple testing using the Benjamini–Hochberg procedure, and pathways with FDR < 0.05 were considered statistically significant.

Differential expression between dormant-like and proliferative cell clusters was performed using the FindMarkers function in Seurat, applying the Wilcoxon rank-sum test. Differential expression between dormant-like and proliferative cells was performed using the sc.tl.rank_genes_groups function in Scanpy, employing the Wilcoxon rank-sum test (method = ‘wilcoxon’). Genes were considered significantly differentially expressed if they met the following criteria: |log_2_FC| ≥ 1 and Benjamini–Hochberg adjusted *p*-value < 0.05.

GO enrichment analysis was performed using the gseapy package based on the hypergeometric distribution. GO biological process terms with a *p*-value ≤ 0.05 and an adjusted *p*-value (Benjamini–Hochberg FDR) ≤ 0.05 were considered statistically significant. Pathway enrichment analysis of drug targets was performed using Enrichr. Three widely used pathway databases, Kyoto Encyclopedia of Genes and Genomes (KEGG), Reactome, and WikiPathways, were considered. The significance thresholds of enrichment results were *p*-value ≤ 0.05 and FDR ≤ 0.05.

All survival analyses were performed using the survival and survminer R packages. The optimal cutoff stratifying patients was determined by the surv_cutpoint function in the survminer R package. Kaplan–Meier curves were generated using the survfit function in the survival R package. Survival distributions were compared using the log-rank test, with *p* < 0.05 considered statistically significant.

For bootstrap resampling (DCA and NRI), 1000 iterations were performed, and 95% confidence intervals were derived from the 2.5th and 97.5th percentiles of the bootstrap distribution. For NRI, risk categories were defined as low, intermediate, and high based on the 33rd and 67th percentiles of the risk scores from the marker-gene model. Statistical significance was assessed using bootstrap *p*-values.

## 5. Conclusions

In summary, this study provides an in-depth insight into the molecular mechanism of CRC dormancy through a combined analysis of bulk and single-cell transcriptomic data. The GMMPS algorithm identified seven dormancy-related energy metabolic pathways, and single-cell analysis further revealed the dormant-like cells and their gene signatures in epithelial, cancer stem, and immune cells. We identified 23 CDAGs that participate in metabolic processes affecting energy supply or substance synthesis in dormant-like cells. These genes could be used to identify individuals whose tumor cells are in a stable dormant state and who have a better clinical prognosis. Targeting these genes to maintain tumor dormancy may represent a promising therapeutic strategy for preventing CRC metastasis and recurrence.

## Figures and Tables

**Table 1:** Data cohorts and their roles in this study.

	GEO ID	Sample Description	Data Type	Reference
Dormancy-related metabolic pathway discovery	GSE114012	Dormant vs. proliferative CRC cell lines (experimentally induced)	Bulk transcriptomic data	[43]
Dormant-like cell selection & signature definition	GSE234804	Primary CRC and liver metastases (biopsies of metastatic sites)	Single-cell data	[44]
Preliminary exploration	GSE193248	*n* = 15 (Engineered organoids; 9 metastatic (proliferative), 6 micrometastatic (dormant-like))	Bulk transcriptomic	[45]
External classification validation	GSE41258	*n* = 253 (67 metastatic: primary tumors with synchronous metastasis at diagnosis, M1; 186 non-metastatic: M0)	Bulk transcriptomic	[46]
	GSE41568	*n* = 133 (94 metastatic: M1; 39 non-metastatic: M0)	Bulk transcriptomic	[47]
Prognostic cutoff determination	GSE41258	*n* = 217 (recurrence-free survival, RFS, subset of the cohort)	RFS	[46]
Prognostic evaluation	TCGA COADREAD	*n* = 564 (progression-free survival, PFS)	PFS	[48]

## Data Availability

The data presented in this study are available in the Gene Expression Omnibus (GEO) at https://www.ncbi.nlm.nih.gov/geo/ (accessed on 18 March 2025), reference numbers GSE114012 and GSE234804, and from The Cancer Genome Atlas (TCGA) Colon and Rectal Cancer (COADREAD) dataset.

## Supplementary Materials

The following supporting information can be downloaded at: https://www.mdpi.com/article/10.3390/ijms27125191/s1.

## Abbreviations

The following abbreviations are used in this manuscript:

CRC	Colorectal cancer
CDAGs	CRC dormancy-associated genes
GMMPS	Gene–Metabolite–Metabolic Pathway–Scoring
GEM	Genome-scale metabolic models
PFS	Progression-free survival
RFS	Recurrence-free survival
