# Supplementary material for: Combined Analysis of Bulk and Single-Cell Transcriptomic Data Reveals Dormancy-Associated Genes in Colorectal Cancer
Source: Int J Mol Sci. 2026 Jun 8;27(12):5191. doi: 10.3390/ijms27125191 (PMC13300251; doi:10.3390/ijms27125191)
Supplement: Supplementary file 1 [file ijms-27-05191-s001.zip › ijms-4317431-supplementary.pdf]

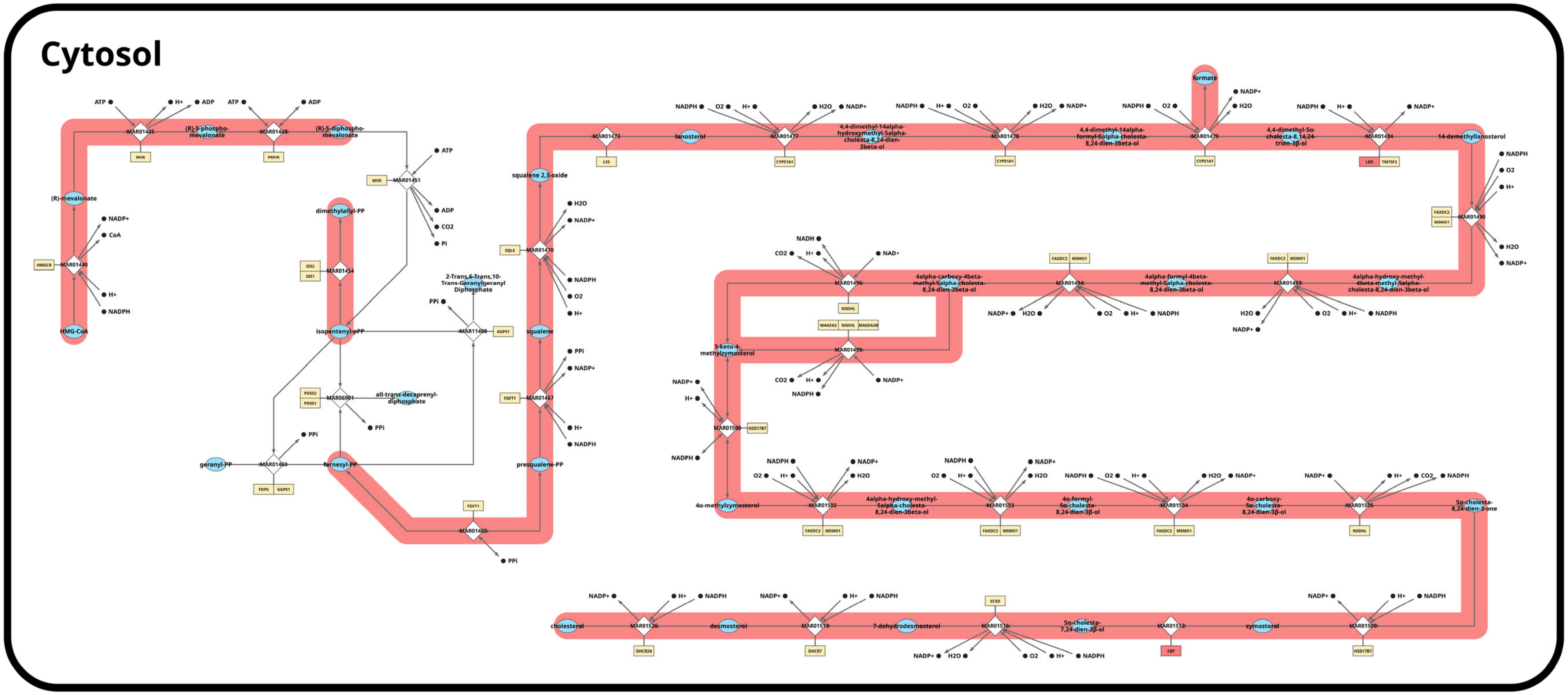

Figure S1. Main part of the Cholesterol biosynthesis 1 (Bloch pathway) pathway. Red nodes are CDAGs.

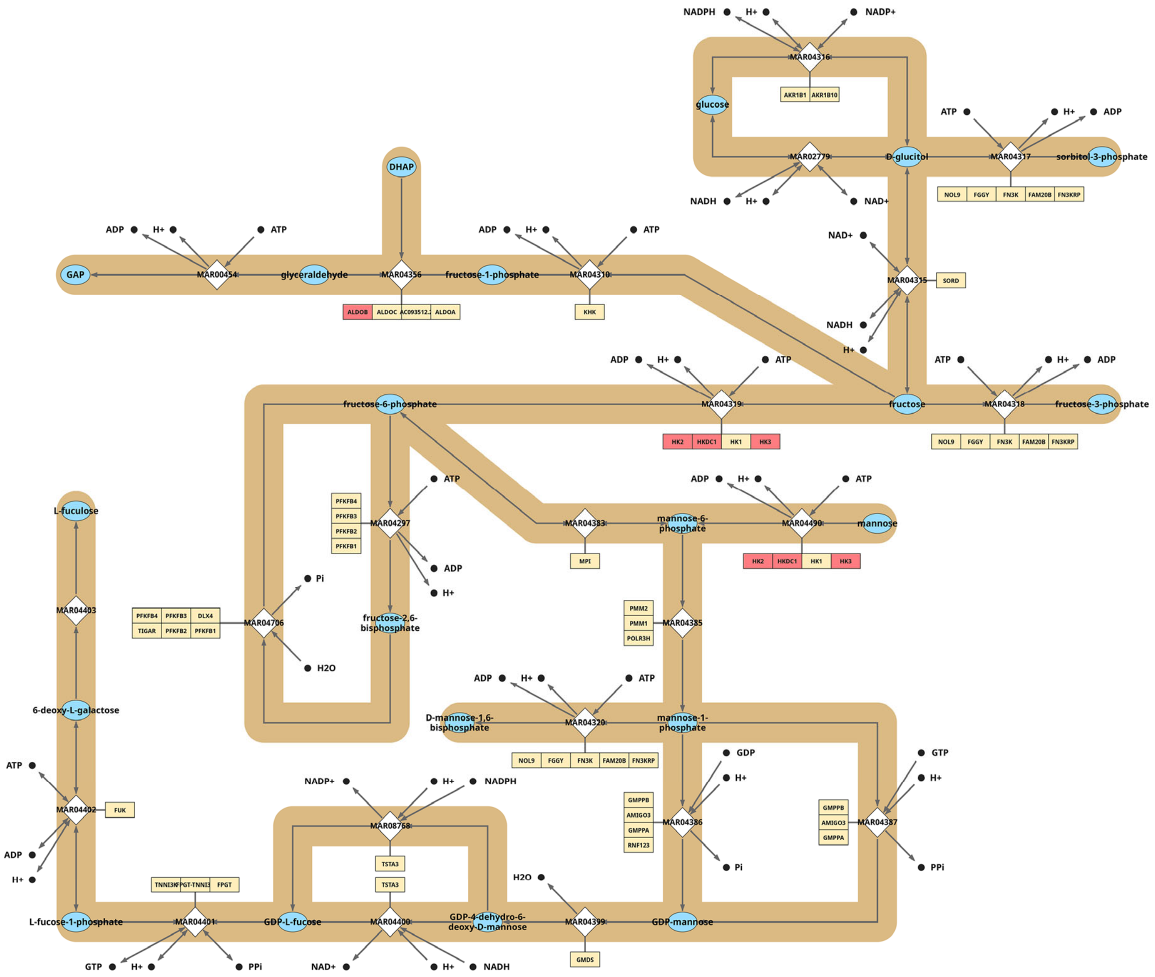

Figure S2. Main part of the Fructose and mannose metabolism pathway. Red nodes are CDAGs.

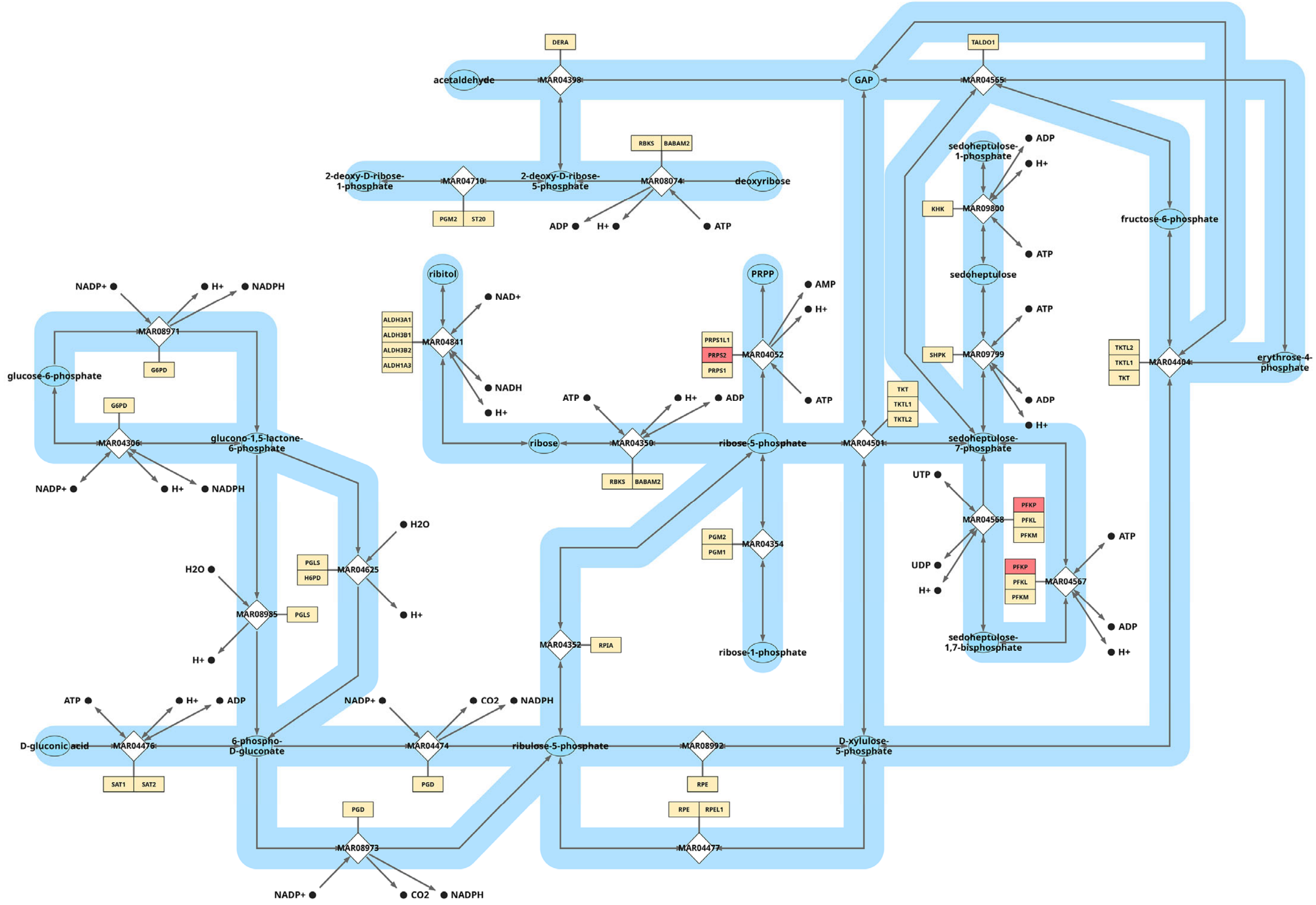

Figure S3. Main part of the Pentose phosphate pathway. Red nodes are CDAGs.

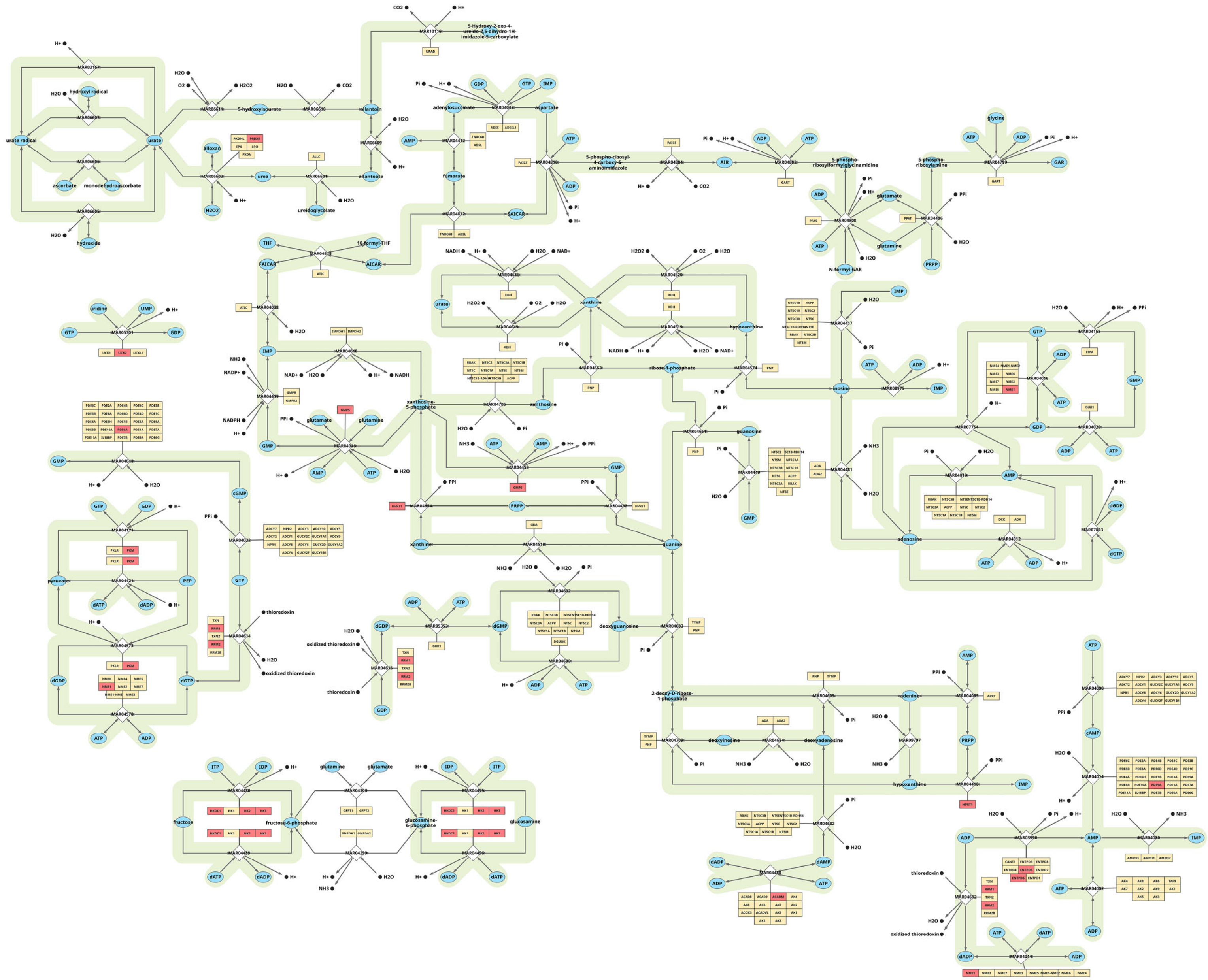

Figure S4. Main part of the Purine metabolism pathway. Red nodes are CDAGs.

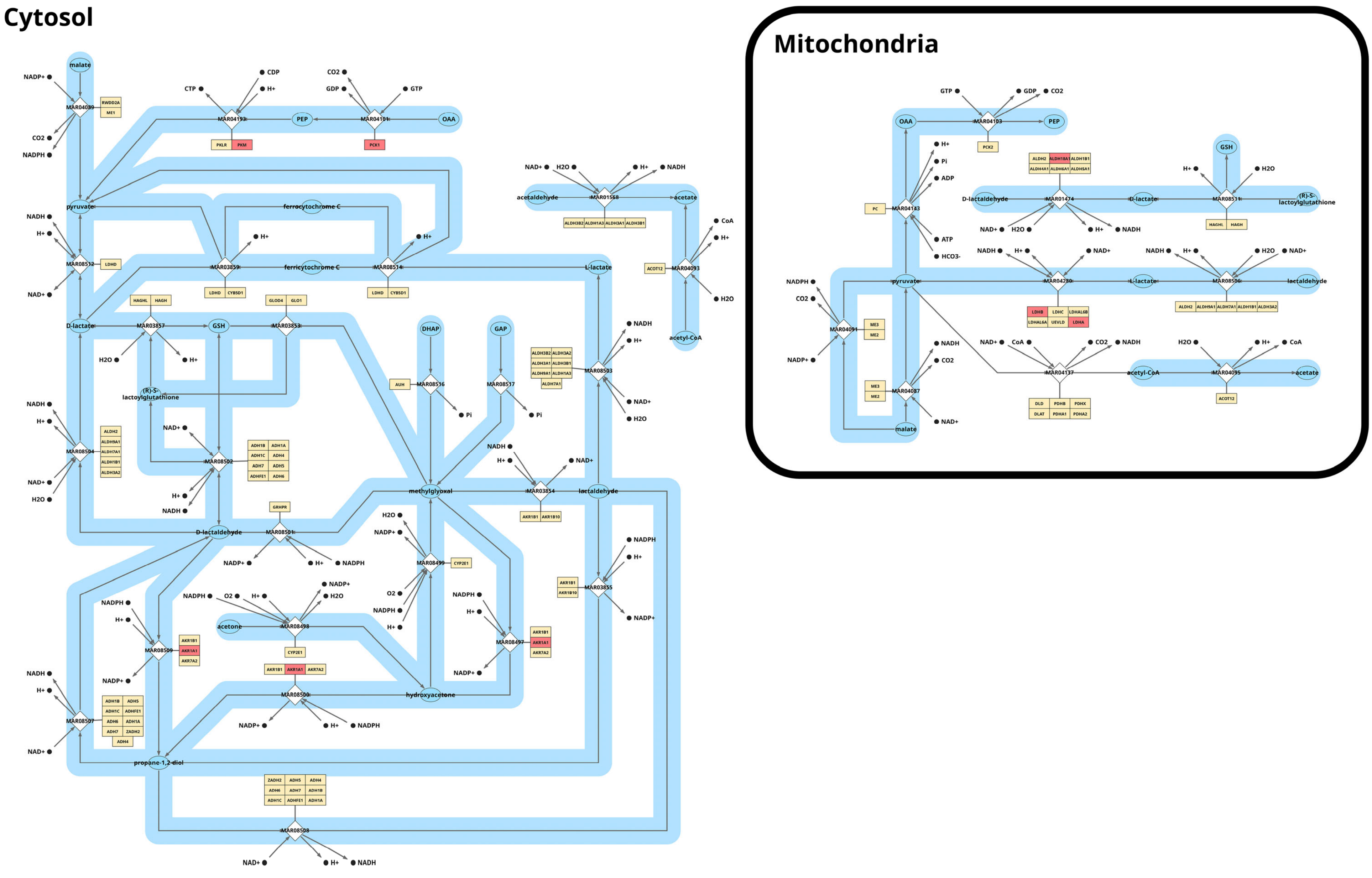

Figure S5. Main part of the Pyruvate metabolism pathway. Red nodes are CDAGs.

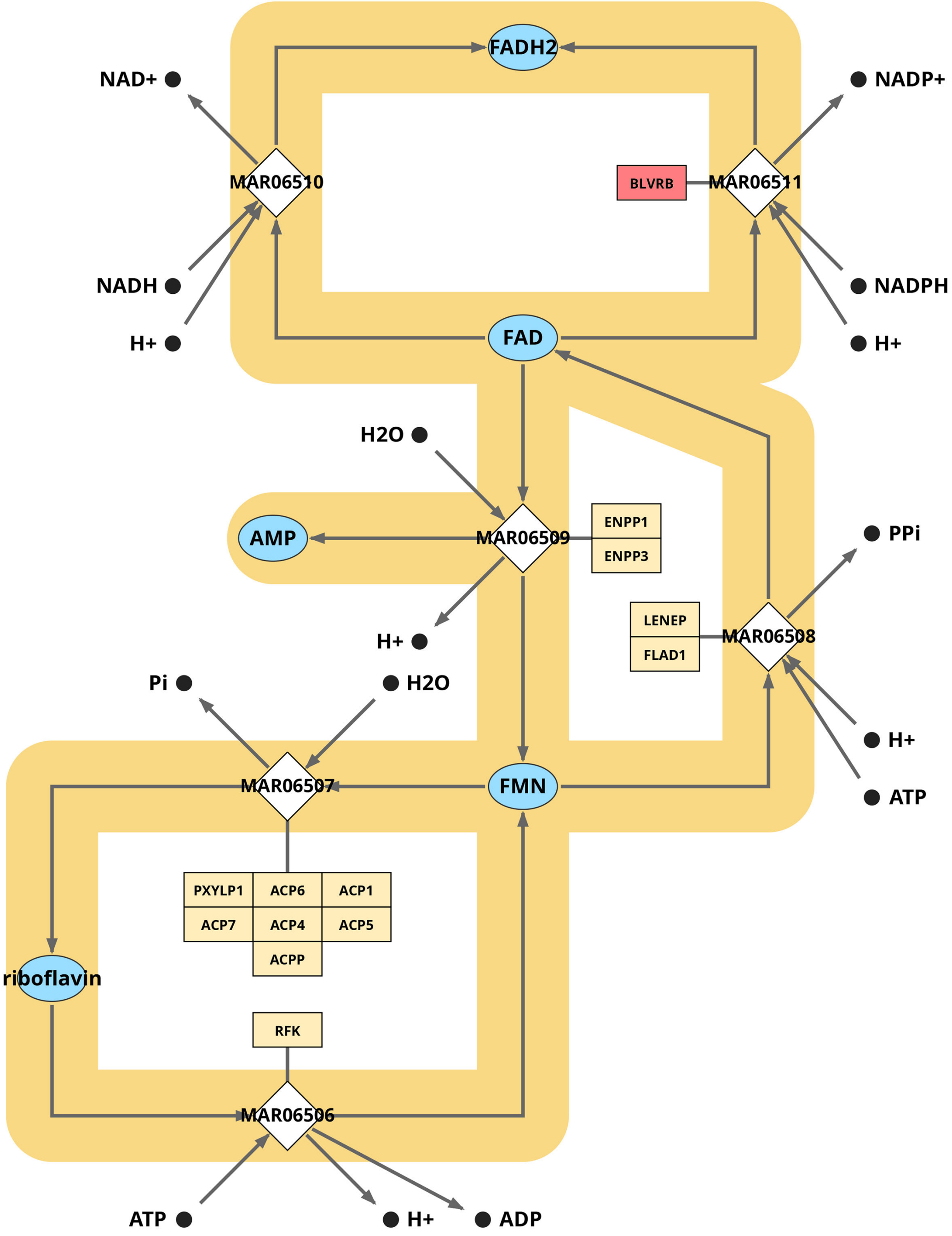

Figure S6. Main part of the Riboflavin metabolism pathway. Red nodes are CDAGs.

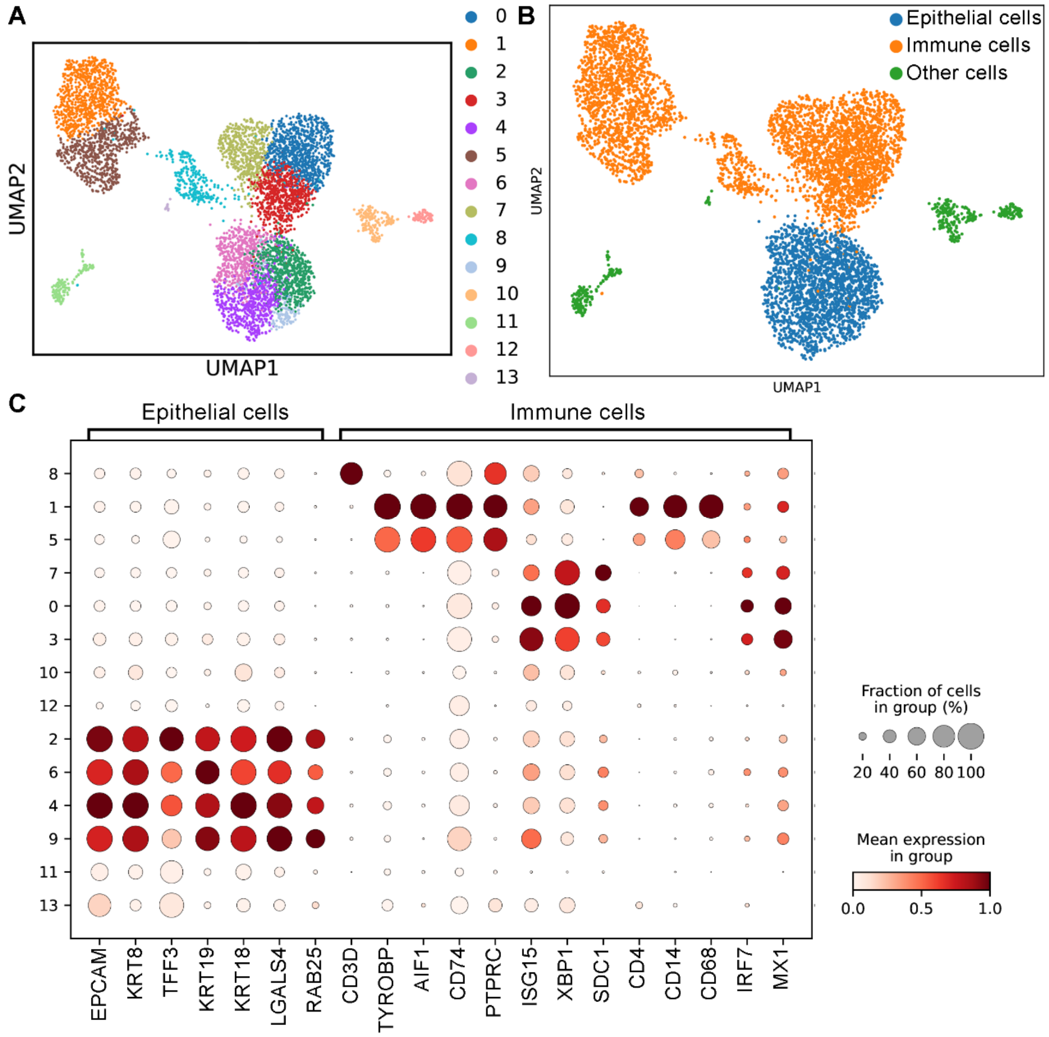

Figure S7. Cell type annotation. (A) Cell clusters. (B) Cell type annotation. (C) Cell type marker genes.

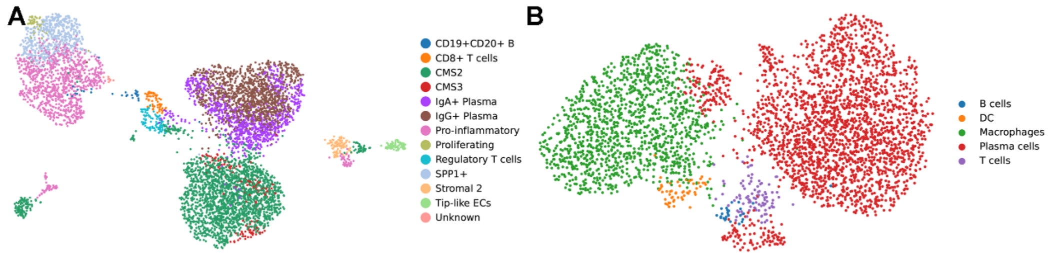

Figure S8. CellTypist cell type annotation. (A) Cell types for CRC. (B) Subtypes for immune cells.

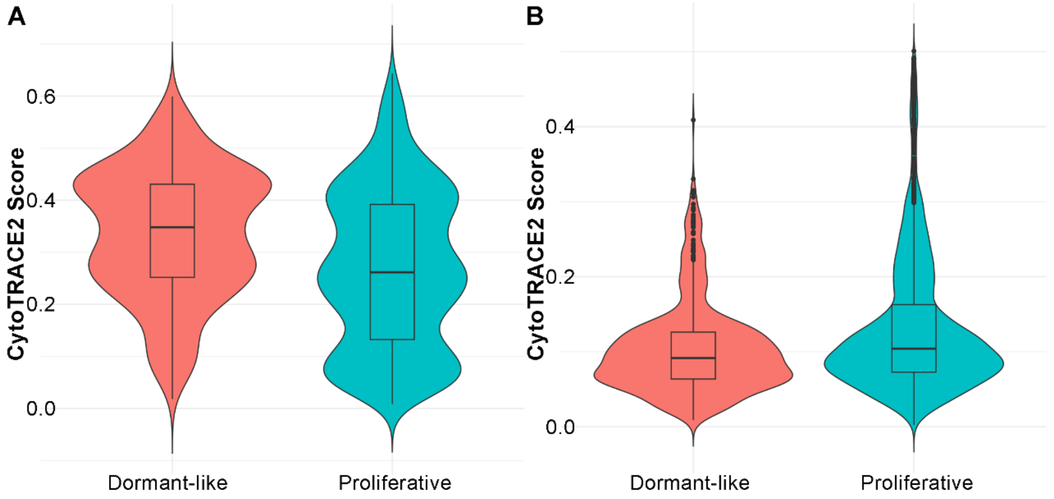

Figure S9. CytoTRACE2 scores comparing dormant-like and proliferative cells for (A) epithelial cells and (B) immune cells.

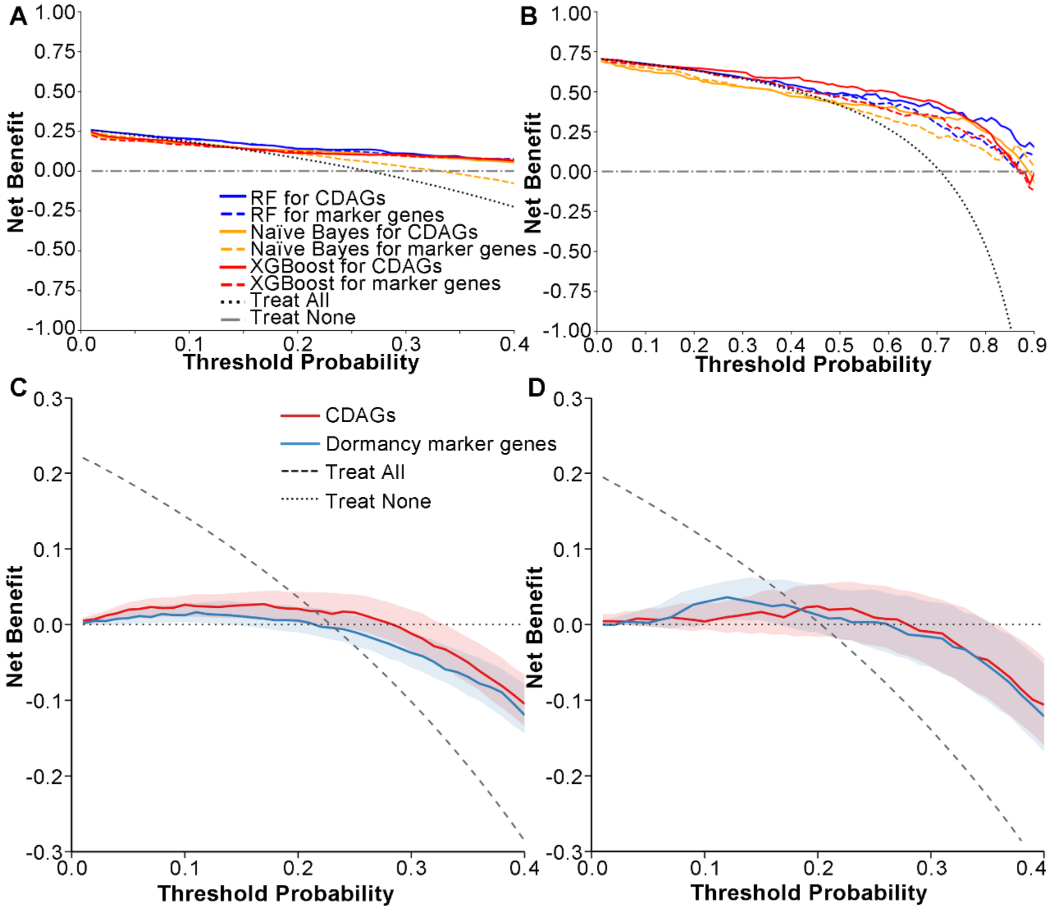

Figure S10. Decision curve analysis comparing CDAGs vs. dormancy marker genes. (A) Classification of metastatic samples (GSE41258, n = 253). (B) Classification of metastatic samples (GSE41568, n = 133). (C) Prognostic prediction of 3-year PFS (TCGA COADREAD, n = 564, truncated at 2,000 days). (D) Prognostic prediction of 3-year RFS (GSE41258, n = 217, truncated at 2,000 days).

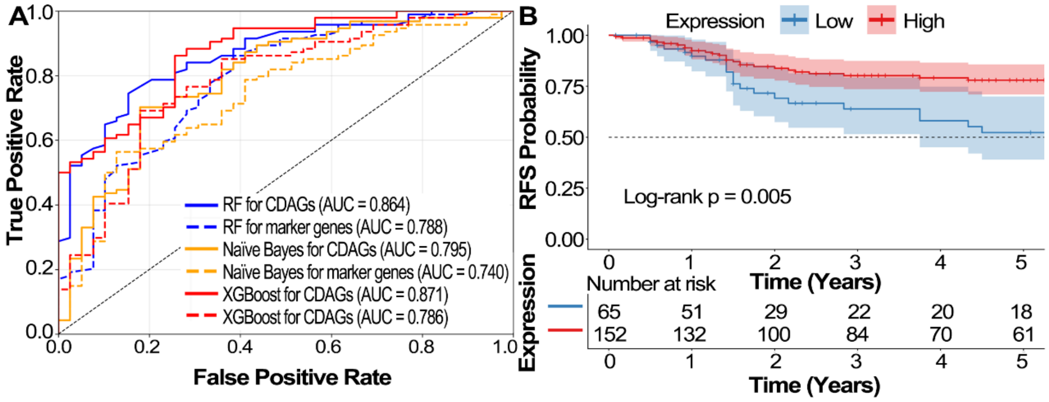

Figure S11. Additional validation of CDAGs in independent cohorts. (A) The ROC curve for five-fold cross-validation of machine-learning models for GSE41568 comparing CDAGs vs. dormancy marker genes. (B) Kaplan-Meier survival curves of CDAGs for GSE41258.

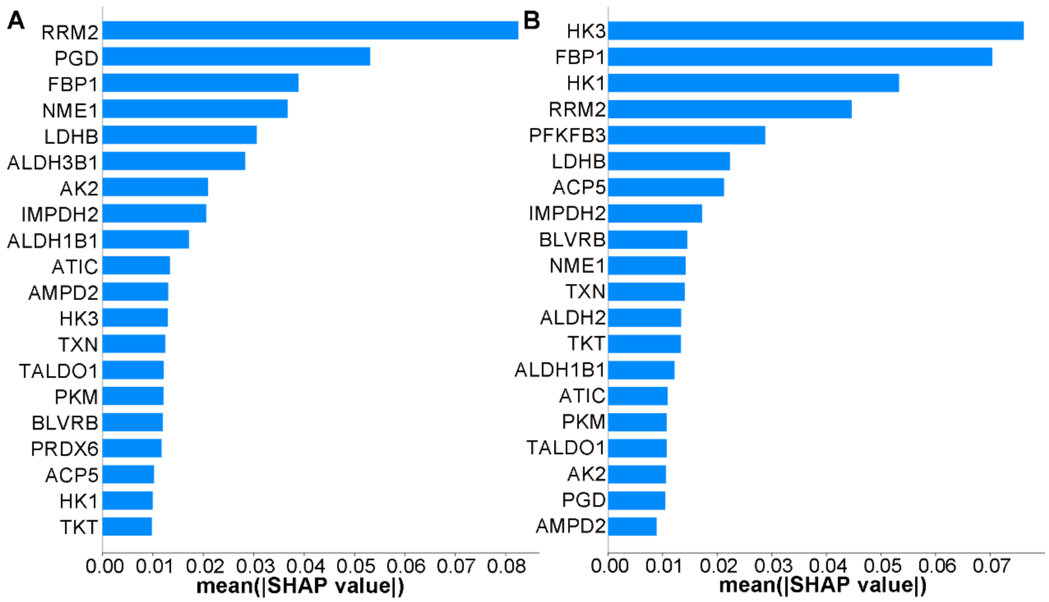

Figure S12. Mean absolute SHAP values of CDAGs in RF classifiers for two independent CRC cohorts: (A) GSE41258 and (B) GSE41568.

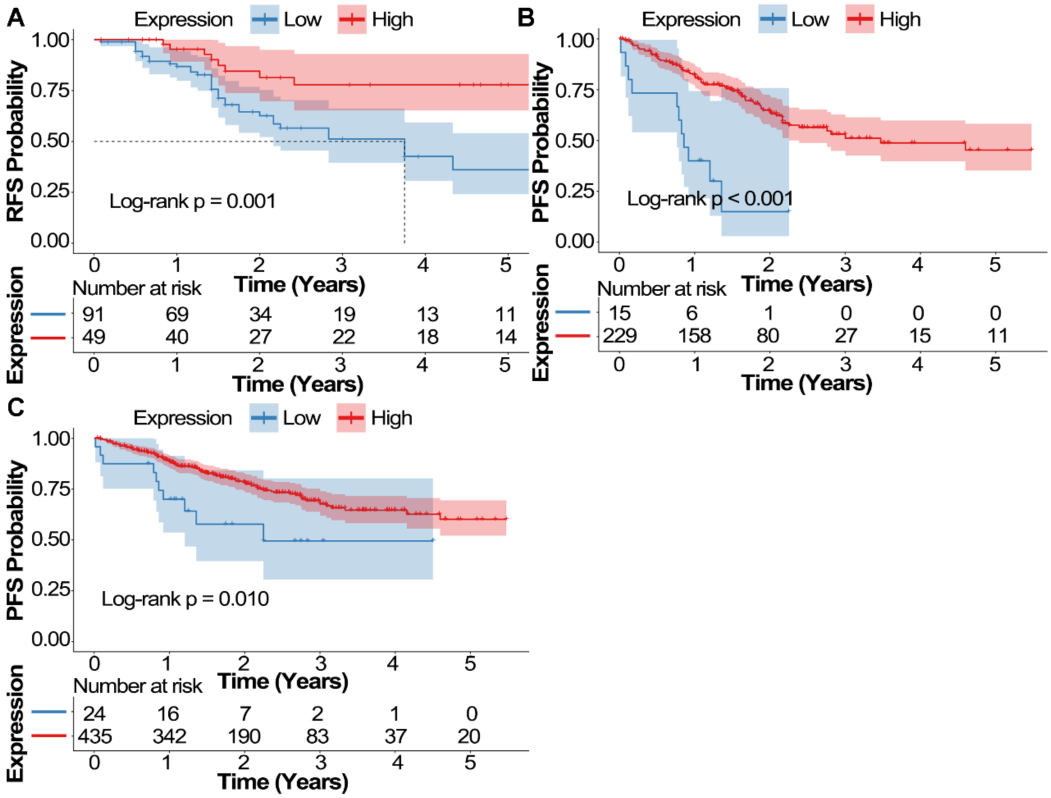

Figure S13. Kaplan-Meier survival curves of CDAGs for GSE41258 in late-stage patients (Stage III-IV, p = 0.001), (B) TCGA COADREAD in late-stage patients (Stage III-IV, p < 0.001) and (C) TCGA COADREAD in those not receiving adjuvant chemotherapy (p = 0.010).

Table S1. Up/down-regulated genes of different dormant-like cells in dormancy related metabolic pathways.

Cell type	Upregulated in dormant-like cells	Downregulated in dormant-like cells
Epithelial cell	*ALDH1B1, ALDH2, TKT, AK2, LDHB, AMPD2, APRT, ATIC, PKM, IMPDH2, NME1, PRDX6, TXN*	*RRM2*
Immune cell		*ALDH3B1, ACP5, FBP1, BLVRB, HK1, PGD, HK3, ALDH2, PFKFB3, TALDO1, TKT, PKM, TXN*

Table S2. Metrics across the five cross-validation folds.

Classifier	Fold	AUC	Accuracy	F1
RF	Fold 1	0.797	0.765	0.500
	Fold 2	0.852	0.863	0.667
	Fold 3	0.863	0.804	0.444
	Fold 4	0.796	0.740	0.316
	Fold 5	0.749	0.780	0.476
Naïve Bayes	Fold 1	0.846	0.804	0.667
	Fold 2	0.846	0.725	0.533
	Fold 3	0.814	0.745	0.606
	Fold 4	0.730	0.740	0.519
	Fold 5	0.763	0.700	0.545
XGBoost	Fold 1	0.820	0.784	0.593
	Fold 2	0.819	0.804	0.583
	Fold 3	0.802	0.765	0.455
	Fold 4	0.865	0.800	0.583
	Fold 5	0.730	0.720	0.462

Table S3. Detailed classification performance of Naïve Bayes and XGBoost for CDAGs versus dormancy marker genes in two independent cohorts.

Cohort	Model	Metric	Difference (95% CI)	p-value
GSE41258	Naïve Bayes	NRI	0.186 (-0.105 to 0.449)	0.184
		IDI	0.187 (0.039 to 0.325)	0.010
		ΔNB at threshold 0.25	0.047 (0.004 to 0.091)	0.035
		ΔNB at threshold 0.30	0.069 (0.021 to 0.118)	0.006
	XGBoost	NRI	0.137 (−0.146 to 0.422)	0.367
		IDI	0.008 (−0.094 to 0.118)	0.913
		ΔNB (all thresholds)	Not significant	>0.05
GSE41568	Naïve Bayes	NRI	0.567 (0.221 to 0.916)	0.002
		IDI	0.145 (−0.008 to 0.310)	0.063
		ΔNB (all thresholds)	Not significant	>0.05
	XGBoost	NRI	0.478 (0.115 to 0.811)	0.008
		IDI	0.147 (−0.007 to 0.295)	0.067
		ΔNB (all thresholds)	Not significant	>0.05

Table S4. Clinical characteristics of patients for survival analysis.

	Variable	N (%)
TCGA COADREAD	Age (mean±SD)	65.9±12.8
	Sex-Male	302 (53.5%)
	Sex-Female	262 (46.5%)
	Stage I	101 (17.9%)
	Stage II	207 (36.7%)
	Stage III	165 (29.3%)
	Stage IV	79 (14.0%)
	Stage Group-Early(I-II)	308 (54.6%)
	Stage Group-Late(III-IV)	244 (43.3%)
	T Stage-T1-2	120 (21.3%)
	T Stage-T3-4	408 (72.3%)
	N Stage-N0	325 (57.6%)
	N Stage-N1	97 (17.2%)
	N Stage-N2	70 (12.4%)
	M Stage-M0	422 (74.8%)
	M Stage-M1	63 (11.2%)
	Radiation-Yes	27 (4.8%)
	Radiation-No	459 (81.4%)
	Subtype-CIN	315 (55.9%)
	Subtype-MSI	60 (10.6%)
	Subtype-GS	55 (9.8%)
	Subtype-POLE	9 (1.6%)
	PFS events-Progression	140 (24.8%)
GSE41258	Age (mean±SD)	63.3±14.7 years
	Age (median[range])	65[19-87] years
	Sex-Male (M)	121 (55.8%)
	Sex-Female (F)	96 (44.2%)
	Stage I	31 (14.3%)
	Stage II	46 (21.2%)
	Stage III	54 (24.9%)
	Stage IV	86 (39.6%)
	Stage Group-Early (I-II)	77 (35.5%)
	Stage Group-Late (III-IV)	140 (64.5%)
	RFS events Recurrence	54 (24.9%)

Table S5. Dormancy marker genes and related literature IDs.

Dormancy-related marker genes	Description	PubMed ID
FBXO8, ***CKB***, *CKM*, *CKMT2*, ***CKMT1A***, ***CKMT1B***, ***CDH1***, *SOX2*, ***PROM1***, ***CASP3***	FBX8 could upregulate CK, E-cadherin, Sox-2, Caspase-3, and some other markers related to tumor cell dormancy	PMID：32796813
BMP7	BMP7 gene (bone morphogenetic protein-7), which belongs to the transforming growth factor-β superfamily and is associated with the dormancy of cancer cells	PMID：38609535
COPS8, ***BHLHE41***, *NR2F1*, ***CDKN1B***	The CSN8-overexpressed HCT116 and DLD-1 cells expressed higher levels of dormant markers (NR2F1, DEC2, p27)	PMID：33261601

Genes that were actively used as dormancy markers are in bold.
